# An overview of technical considerations when using quantitative real-time PCR analysis of gene expression in human exercise research

**DOI:** 10.1371/journal.pone.0196438

**Published:** 2018-05-10

**Authors:** Jujiao Kuang, Xu Yan, Amanda J. Genders, Cesare Granata, David J. Bishop

**Affiliations:** 1 Institute for Health and Sport, Victoria University, Melbourne, Victoria, Australia; 2 College of Health and Biomedicine, Victoria University, Melbourne, Victoria, Australia; 3 Department of Diabetes, Central Clinical School, Monash University, Melbourne, Victoria, Australia; 4 School of Medical and Health Sciences, Edith Cowan University, Joondalup, Western Australia, Australia; University of Helsinki, FINLAND

## Abstract

Gene expression analysis by quantitative PCR in skeletal muscle is routine in exercise studies. The reproducibility and reliability of the data fundamentally depend on how the experiments are performed and interpreted. Despite the popularity of the assay, there is a considerable variation in experimental protocols and data analyses from different laboratories, and there is a lack of consistency of proper quality control steps throughout the assay. In this study, we present a number of experiments on various steps of quantitative PCR workflow, and demonstrate how to perform a quantitative PCR experiment with human skeletal muscle samples in an exercise study. We also tested some common mistakes in performing qPCR. Interestingly, we found that mishandling of muscle for a short time span (10 mins) before RNA extraction did not affect RNA quality, and isolated total RNA was preserved for up to one week at room temperature. Demonstrated by our data, use of unstable reference genes lead to substantial differences in the final results. Alternatively, cDNA content can be used for data normalisation; however, complete removal of RNA from cDNA samples is essential for obtaining accurate cDNA content.

## Introduction

Fluorescence-based quantitative real-time polymerase chain reaction analysis of gene expression is an important measure in many fields of biological research. While the technique is also abbreviated as RT-PCR or qRT-PCR, in the Minimal Information for Publication of Quantitative Real-Time PCR Experiments (MIQE) guidelines, it was proposed to use the abbreviation qPCR to avoid confusion in the literature [1]. qPCR is a common approach to measure the expression of target genes in a wide range of samples from many sources, such as tissues, blood, and cultured cells originating from humans, animals, plants, and bacteria. The qPCR technique was pioneered in the early 1990s [2]; however, the application of qPCR to explore exercise-induced changes in gene expression in human skeletal muscle is relatively recent, with the first published report in 2000 [3]. While the results of research using qPCR have greatly increased our understanding of changes in gene expression occurring in skeletal muscle in response to exercise, the increased use of qPCR techniques by exercise scientists without a strong molecular biology background means that important quality control elements may sometimes be overlooked. This has implications for the quality of the data obtained, and may unintentionally lead to incorrect interpretation of the results.

**Fig 1:**
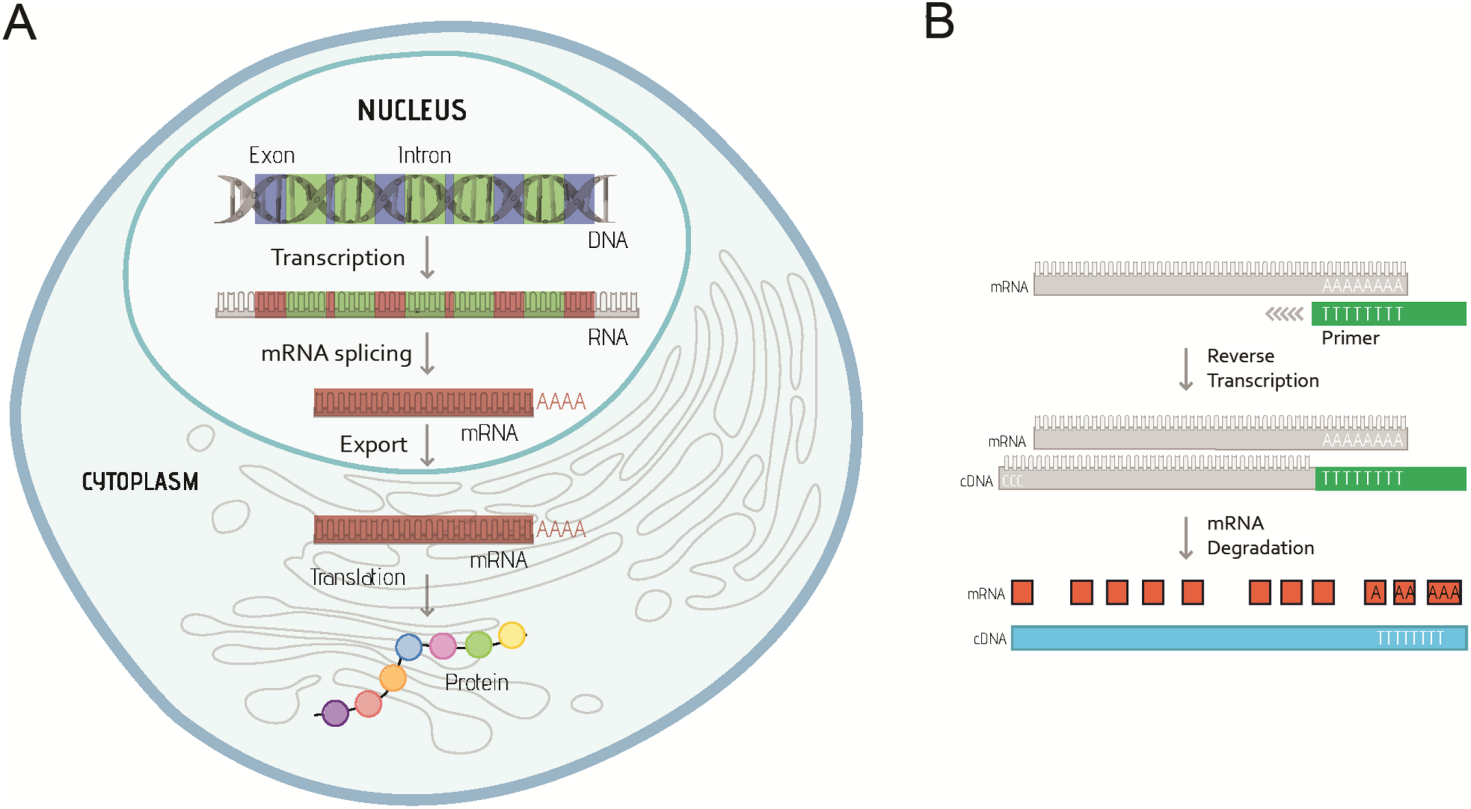
A. Summary of the key steps leading from gene to protein expression in eukaryotes. DNA is first transcribed into RNA, then processed to mRNA after removing the noncoding regions (introns, green) and splicing the coding regions (exons, red) together. The spliced mRNA (red) is then exported to the cytoplasm to produce the protein molecule. B. Outline of procedures for first strand complementary DNA (cDNA) synthesis from messenger RNA (mRNA) using short sequences of deoxy-thymidine nucleotides (oligo-dT primers, green). After annealing of Oligo-dT primers to the mRNA sequence (grey), the RNA-directed DNA polymerase, reverse transcriptase, is able to synthesise cDNA strand (blue), as well as degrade mRNA (red) from the hybrid molecule.

Gene expression is regulated at different stages, including: transcription (copy genetic information from genomic DNA into RNA), post-transcriptional modification (convert primary transcript RNA into messenger RNA [mRNA]), translation (produce polypeptide chains based on mRNA), and post-translation modification (chemical changes of protein after translation) (Fig 1A). When using qPCR to quantify gene expression by measuring the level of mRNA, total RNA needs to be extracted from the experimental sample and the mRNA is required to be converted into complementary DNA (cDNA) through a process called reverse transcription (Fig 1B), and then used as the template for the qPCR reaction.

The basis of qPCR is to monitor the process of DNA polymerase-driven DNA amplification, which is known as the polymerase chain reaction (PCR), in “real-time”. In a PCR reaction, a thermostable DNA polymerase enzyme is used to synthesise new strands of DNA complementary to the target DNA sequence. In the reaction, this enzyme is mixed with the DNA template (starting genetic material that contains the target sequence), forward and reverse primers (short pieces of single-stranded DNA designed to bind to target DNA sequence and allow DNA synthesis in both directions), and nucleotides (single units of the DNA bases, also known as deoxynucleotide triphosphates [dNTPs]). The reaction proceeds through three cyclically repeated reactions: denaturing (strand separation), annealing (primer binding), and extending (new strand synthesis). At the end of the PCR reaction, the target sequence will be amplified in billions of copies (PCR amplicons). Unlike traditional PCR, qPCR is able to detect the amplification of the PCR amplicons at the end of each amplification cycle by using a fluorescent dye system and a thermocycler with fluorescence-detection capability.

qPCR is fast and easy to perform compared to other RNA quantification methods, such as northern blotting and *in situ* hybridization. In addition, the detection method of qPCR is more sensitive and specific compared to the other assays [4]. It, therefore, provides scientists with the ability to perform accurate high-throughput mRNA quantification over a wide dynamic range [5].

**Fig 2:**
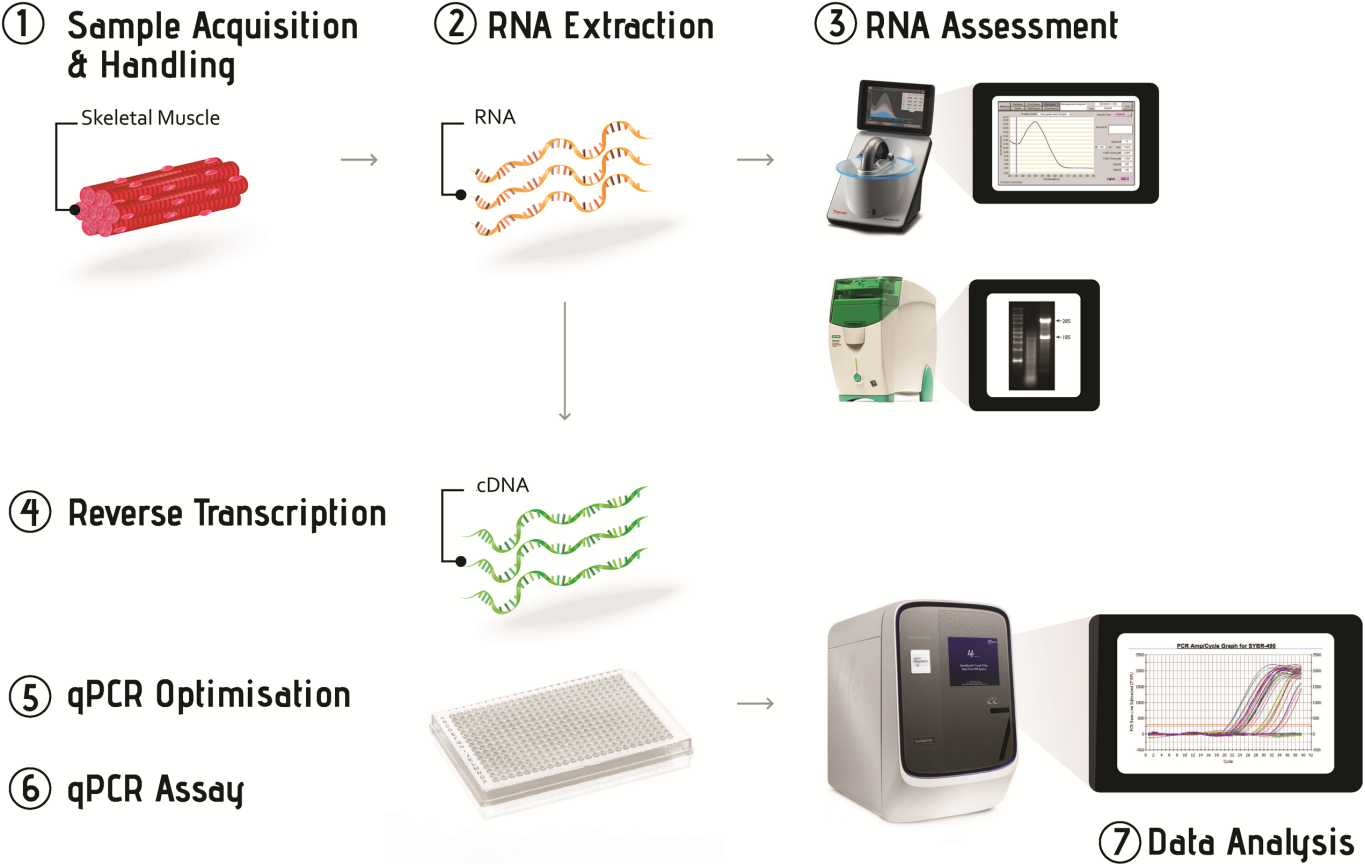
The sequential stages of the quantitative real-time PCR workflow. Skeletal muscle samples are cleaned quickly and snap-frozen in liquid nitrogen after the muscle biopsy, and then stored at -80°C until RNA extraction. RNA is extracted using TRIzol or another Tri-reagent, and assessed for concentration and quality. RNA that passes the quality test is used to synthesise cDNA, which is used as template in the final qPCR assay. The conditions of the final qPCR assay must be optimised for each experiment, and the data should be processed using the correct analysis methods.

The workflow of gene expression analysis using the qPCR technique is based around seven key steps: (1) acquisition and handling of the experimental samples; (2) extraction of total RNA from experimental samples; (3) assessment of RNA concentration and quality; (4) synthesis of cDNA from extracted total RNA through reverse transcription; (5) optimising conditions for the qPCR assay; (6) running the qPCR reaction under optimised conditions to measure the expression level of target genes; and (7) data analysis using appropriate normalisation methods (Fig 2).

Even though qPCR is considered one of the most effective methods to quantify gene expression, capable of detecting mRNA with low expression levels [6], the quality of the results can be affected by variations in how the workflow described above is implemented by different laboratories. This has led researchers to express concerns regarding the results obtained from poorly-designed methods with inadequate quality control steps [1, 7]. The most common issues include sample handling and preparation, quality of RNA extracted, use of inappropriate reference genes, and normalisation of the data [1, 8].

As previously stated, although qPCR can be applied to cell culture and animal studies, we only focused on the application of this technique to human skeletal muscle samples in the current study. The aim was to describe and discuss in detail the key steps of the qPCR workflow. We have also tested how variations in some key aspects of experimental design and execution may affect the results.

## Step 1: Sample acquisition and handling

Muscle sample acquisition and handling prior to total RNA extraction can potentially introduce variation to the final results, as mRNA expression can be induced or repressed by inappropriate sample collection and processing [1, 9]. The most commonly used method is to process the muscle samples immediately after taking a muscle biopsy, and to quickly snap-freeze in liquid nitrogen. The rate of RNA degradation varies in different tissues, and also depends on preparation and storage conditions. Research has shown that good RNA stability was maintained for up to five hours at room temperature in human lung tissue [10], and others have reported that RNA from tonsil and colon tissue was stable for several hours on ice [11]. However, another study has shown that incubation of freshly-obtained mouse liver tissue at 37°C for 4 hours resulted in extensive RNA degradation and strongly affected the measured levels of some mRNAs [12]. Our recommendations for sample acquisition and handling are listed in Box 1.

**Box 1**

To prevent RNA degradation and to preserve mRNA profiles, rapid sample handling is recommended. Once obtained, muscle samples should be quickly cleaned of excess blood, fat, and connective tissue, so that non-skeletal muscle cell contamination is reduced. Samples should then be snap-frozen in liquid nitrogen immediately and remain frozen until RNA extraction with as little manipulation as possible. Nonetheless, if for some reason these recommended procedures are not followed, it is worth checking the RNA concentration and quality as the samples may still be viable (see Experiment 1).

## Step 2: RNA extraction

In human studies, we typically obtain around 100 to 300 mg (wet weight) of skeletal muscle using a Bergström biopsy needle with manual suction applied [13], which will be utilised for multiple analyses. Thus, an efficient and reliable RNA extraction protocol is essential for working with a limited amount of starting material. In our laboratory, we have found that TRIzol (or Tri-reagent) is required for successful RNA extraction from human muscle samples. TRIzol is a monophasic solution of phenol and guanidinium isothiocyanate that simultaneously solubilises biological material (e.g., tissues and cells of human, animal, plant, yeast or bacterial origin) and denatures proteins. After solubilisation, RNA is separated from protein and DNA in an aqueous phase created by the addition of chloroform [14]. RNA extraction can then be performed as a TRIzol-based extraction, in which the aqueous phase containing RNA is manually transferred to a clean tube and precipitated using 2-propanol or ethanol. An alternative is to use commercial extraction kits that also contain a TRIzol lysis step, in which RNA is purified by passing the RNA-containing aqueous phase through an RNA-binding spin column. Although some kits (e.g., the RNeasy Plus Universal Mini Kit from Qiagen) recommended using ethanol to precipitate RNA, our experiments indicate that replacing ethanol with 2-propanol to precipitate RNA increased the RNA concentration (see Experiment 2). Tri-reagent based extraction methods are usually cheaper and can provide a higher RNA yield than column-based methods. However, commercially-available extraction kits with spin columns are able to provide RNA of higher purity, which is required for applications such as microarray and RNA sequencing [15]. Our recommendations for RNA extraction are listed in Box 2.

**Box 2**

For efficient RNA extraction from human skeletal samples, we recommend lysing muscle samples with TRIzol or another Tri-reagent, and to perform the RNA precipitation with 2-propanol rather than ethanol (see Experiment 2). It is required by the MIQE guidelines to provide detailed information on how the RNA extraction was performed, including experimental procedure, as well as the instruments and kit used [1].

## Step 3: RNA assessment

Assessment of both RNA concentration and purity in extracted samples can be carried out using a UV/VIS spectrophotometer. Absorbance at 260 nm (*A*_260_) gives a specific measurement of nucleic acid concentration, and the absorbance at 280 nm (*A*_280_) and 230 nm (*A*_230_) measures protein and background absorption, respectively, as an indication of possible contaminants. It is important to keep in mind that the 260 nm wavelength detects both RNA and DNA, and the presence of genomic DNA contamination could lead to an over-estimation of RNA concentration. An *A*_260_ reading of 1.0 equals 40 μg·mL^−1^ of RNA or 50 μg·mL^−1^ of double stranded DNA. In general, an *A*_260_/*A*_280_ ratio of 1.8 to 2.1 at pH 7.5 indicates very pure RNA, and a ratio greater than 1.8 is considered an acceptable indicator of good quality RNA [16, 17]. Pure RNA should also give an *A*_260_/*A*_230_ ratio of 2 or slightly above; however, there is no acceptable lower limit of this ratio, as it is not clear which contaminants contribute to a low *A*_260_/*A*_230_ ratio [17, 18]. Moreover, previous research and our data have both shown that there was no significant correlation between *A*_260_/*A*_230_ and qPCR amplification efficiency [18] (S1 Fig).

**Fig 3:**
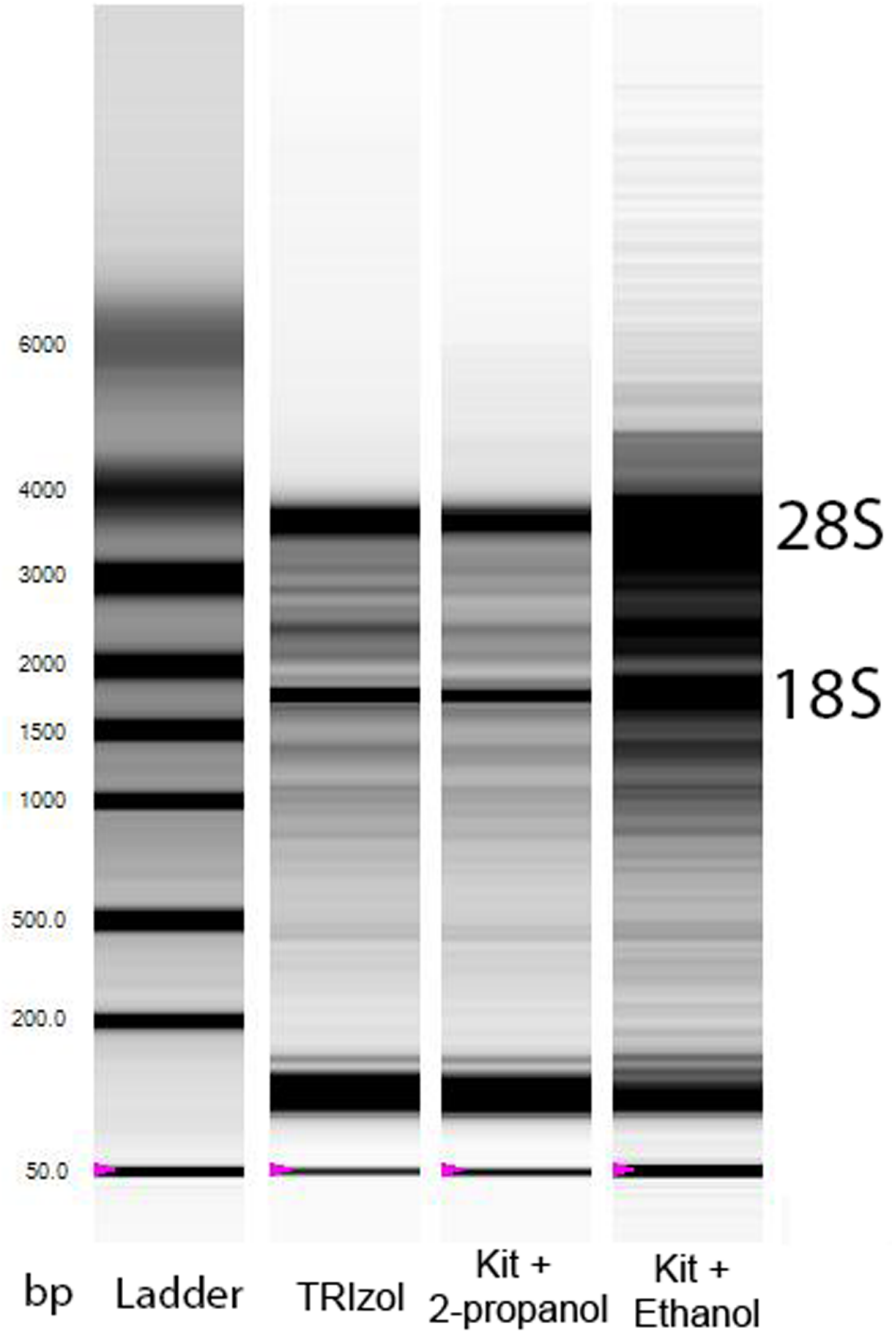
Densitometric gel-like image (virtual gel image) of RNA samples extracted from the same muscle sample using different methods. The images were generated by a Bio-Rad Experion microfluidic gel electrophoresis system. The first lane is the molecular size marker (ladder), which indicates the approximate size of molecules on the gel. For RNA samples extracted using TRIzol (Lane 2) or an extraction kit containing a Tri-reagent lysis step and precipitating RNA using 2-propanol (Lane 3), 28S and 18S rRNA are clearly visible as two sharp bands. For RNA samples extracted using an extraction kit containing a Tri-reagent lysis step and precipitating RNA using ethanol (Lane 4), 28S and 18S rRNA appear as a smear (indicating the accumulation of low molecular weight components) rather than sharp bands. The location of 28S and 18S rRNA is indicated beside the gel image.

While the absorbance ratio of *A*_260_/*A*_280_ does provide an indication of RNA purity, an assessment of RNA quality (or RNA integrity, or intactness of RNA) is still required as the reliability of the qPCR to accurately detect changes in gene expression is affected by degraded RNA [19]. The traditional way to assess RNA quality is to separate the RNA sample in an agarose gel and to visualise with a fluorescent dye. Two sharp bands representing large and small subunits of ribosomal RNAs (28S and 18S), with the intensity of 28S being about twice that of 18S, indicate that the RNA is intact (Fig 3).

In 2005, Imbeaud et al. [20] introduced RNA quality assessment using a microcapillary electrophoresis system from an Agilent bioanalyzer (Agilent Technologies, USA), which measures fluorescence of a fluorophore bound to very small amounts of RNA. In recent years microcapillary electrophoresis systems have become the standard method for RNA quality assessment, and we used a similar system from Bio-Rad Laboratories (Hercules, CA), the Experion automated electrophoresis system, to evaluate the quality of our RNA samples. Both the Agilent bioanalyzer and Experion automated electrophoresis system are widely accepted systems for the assessment of RNA quality [1, 15, 16]. During RNA degradation ribosomal RNAs decrease in size, which leads to an increased accumulation of low molecular weight components.

Manufactures of the Agilent bioanalyzer first introduced an RNA Integrity Number (RIN) algorithm analysis to standardise RNA quality assessment. Approximately 1300 total RNA samples from different tissues were analysed and categorised manually to develop the RIN tool, which is a numbering system from 1 to 10 (which 1 being the most degraded and 10 the most intact). For different downstream applications, there are different recommendations for the minimal RIN values required. For example, RNA sequencing by Illumina recommends using RNA sample with RIN values greater than 8. The Experion automated electrophoresis system generates an RNA Quality Indicator (RQI, from 1 to 10) for each RNA sample by comparing the plot of results from the separation of a sample to a series of standardised degraded RNA samples. Based on this system, the RNA sample is considered to be sufficiently intact if the RQI is greater than 7. Our recommendations for RNA assessment are listed in Box 3.

**Box 3**

Although often absent from exercise studies reporting qPCR results, it is required by the MIQE guidelines to detail the methods and instruments used for RNA quantification and quality measurements, and to report the results (e.g., RNA concentration and RQI; see Experiment 1). It is also advised to use a single method to measure RNA concentration and quality for all the samples in a single study [1]. Based on our results, we recommend using other parameters to assess RNA quality, such as the intactness of RNA with an automated electrophoresis system or gel, rather than the *A*_260_/*A*_230_ ratio.

### Assessment of factors affecting RNA concentration and quality

To assess factors that might affect RNA concentration and quality, muscle samples taken with a Bergström needle from the vastus lateralis of male volunteers (see Materials and methods) were deliberately treated contrary to common recommendations to mimic practices that may affect RNA quality.

#### Experiment 1

To assess the influence of muscle sample handling, four muscle biopsy samples were blotted on filter paper to remove blood before being immediately snap-frozen in liquid nitrogen and then stored at -80°C. Each sample was subsequently divided into three portions (15 to 20 mg each) in a -20°C cold chamber before being returned to -80°C storage. One set of samples (n = 4) was removed from storage at -80°C and left at room temperature for 10 min before RNA extraction (Thaw). Another set (n = 4) was removed from -80°C and thawed at room temperature for 5 min and then frozen on dry ice; this freeze-thaw cycle was repeated three times (Freeze thaw). Another set of samples (n = 4) was removed from -80°C and processed immediately for RNA extraction and was used as a positive control (Good practice).

For samples that were freeze-thawed three times, the yield of RNA was the highest among all the combinations (*P* = 0.031, Table 1). All RNA samples achieved an RQI above 7.8, which indicates the RNA was sufficiently intact with all three handling procedures (Table 1). All of our samples recorded an *A*_260_/*A*_280_ from 1.6 to 1.9, indicating the protein contamination was low (Table 1). The *A*_260_/*A*_230_ ratio was highly variable between samples; however, there is no acceptable lower limit for this ratio.

**Table 1:** RNA concentration, yield, and quality with different sample handling procedures.

RNA extraction	Median of RNA concentration (Range) ng RNA per μL	Median of RNA yield (Range) ng RNA per mg muscle	RQI score > 7	*A*_260_/*A*_280_ range	*A*_260_/*A*_230_ range
Good practice*	279.5 (197.5–340.0)	439.4 (318.5–505.0)	100%	1.8–1.9	0.6–1.2
Thaw*	200.3 (119.0–268.5)	545.4 (324.5–676.9)	100%	1.6–1.8	0.3–1.5
Freeze thaw*	236.5 (166.5–259.5)	778.7 (675.0–949.4)	100%	1.7–1.8	0.4–1.3

* Four biological repeats were tested

Mishandling of muscle samples prior to extraction, including muscle thawing and freeze-thaw cycles, did not have a negative impact on RNA quality. This result suggests that it is possible to extract high-quality RNA from muscle samples even if they are not handled as typically recommended. However, this result may not be universally applied to other analyses of muscle samples, such as western blotting and enzyme activity assays, as well as to all mishandling actions. For example, the manipulations we performed with the muscle samples took place over only a short time-span, and it has been reported that RNA is not viable in tissues that have been defrosted for more than 24 hours [21]. Thus, despite our results, researchers should still be cautious with sample acquisition and handling (see our recommendations in Box 1).

#### Experiment 2

We tested two popular RNA extraction protocols, plus a modified protocol, using four frozen skeletal muscle samples (each 15 to 20 mg) that were frozen immediately in liquid nitrogen and then stored at -80°C. These extraction protocols were: a) TRIzol—using TRIzol reagent from Invitrogen (Thermo Fisher Scientific, Waltham, USA); b) Kit + 2-propanol—RNeasy Plus Universal Mini Kit from Qiagen (Valencia, USA) using 2-propanol to precipitate RNA; and c) Kit + ethanol—RNeasy Plus Universal Mini Kit from Qiagen using ethanol to precipitate RNA, as per the manufacturer’s instructions. As described above, we found that replacing ethanol with 2-propanol when using the RNeasy Plus Universal Mini Kit from Qiagen increased the RNA yield; therefore, we incorporated this modification in our laboratory protocol.

We found RNA yield was much lower when using ethanol to precipitate RNA (P = 0.007 compared to other extraction methods), and one sample out of four did not pass the RNA quality test (RQI = 4.2, cut-off RQI < 7) (Table 2). A virtual gel image of RNA samples is shown in Fig 3 as an example. Sharp, clear 28S and 18S rRNA bands were only seen in samples extracted using TRIzol and the modified kit protocol, but not when extracted with the Kit using ethanol. This result shows that if using the RNeasy Plus Universal Mini Kit protocol for human skeletal muscle RNA extraction, it is advised to use 2-propanol instead of ethanol so as to increase the overall RNA yield and quality, which is critical for subsequent qPCR analyses (see our recommendations in Box 2).

**Table 2:** RNA concentration, yield, and quality with different RNA extraction protocols.

RNA extraction	Median of RNA concentration (Range) ng RNA per μL	Median of RNA yield (Range) ng RNA per mg muscle	RQI score > 7	*A*_260_/*A*_280_ range	*A*_260_/*A*_230_ range
TRIzol*	355.3 (308.0–542.0)	683.1 (616.0–1084.0)	100%	1.7–1.8	0.3–0.5
Kit + 2-propanol*	279.5 (197.5–340.0)	439.4 (318.5–505.0)	100%	1.8–1.9	0.6–1.2
Kit + ethanol*	27.3 (22.0–40.5)	84.8 (59.0–122.7)	75%	0.8–1.1	0.3–0.6

* Four biological repeats were tested

The Tri-reagent based protocol (TRIzol) gave a higher RNA yield compared to the spin column-based protocol (Kit + 2-propanol) (*P* = 0.021, Table 2). All samples were shown to be intact (RQIs of 8.0 to 9.4), according to the recommended system cut-off value (RQI > 7). The difference in RNA yield is consistent with a previous report [15]. Therefore, to extract RNA from human skeletal muscle for the purpose of qPCR analysis, either method is recommended. The choice of extraction method can be decided based on the user’s experience, as well as intended downstream applications of the RNA samples. For example, experienced researchers can choose to use a Tri-reagent based protocol to prepare RNA for qPCR analyses as it is more technically challenging to avoid phenol contamination. When high-quality RNA is required for certain applications, such as RNA sequencing, a spin column based RNA extraction kit is necessary. Another advantage of RNA extraction kits, such as the RNeasy Plus Universal Mini Kit, is that they include a simple on-column genomic DNA elimination step, which removes genomic DNA that could interfere with qPCR if the primers are not specific to cDNA. As a result, we have chosen to use the RNeasy Plus Universal Mini Kit with 2-propanol for RNA extraction in our laboratory (and for the subsequent manipulations reported in this manuscript).

#### Experiment 3

RNA handling and storage is another element for successful qPCR application, as it is important to avoid and inhibit RNase when working with RNA. RNase is a small enzyme that promotes the degradation of RNA into smaller components, and it is found in fluids (such as tears, saliva, and mucus), flaked skin, and hairs.

From the RNA samples extracted using the RNeasy Plus Universal Mini Kit with 2-propanol (Intact RNA), we took a subset of these samples and tried to degrade the RNA by handling without gloves, leaving at room temperature in closed 1.5 mL tubes for 7 days, and pipetting with general-use pipettes and non-barrier tips (Degraded RNA). Two Intact RNA samples were also treated with RNase A (10 μg/mL as a final concentration) and were used as negative controls (RNase Treated). Surprisingly, neither the RNA concentration nor the RNA quality was affected in the ‘Degraded RNA’ sample, when compared with the ‘Intact RNA’ sample (Table 3).

**Table 3:** RNA concentration and quality of different storage methods.

RNA Sample	Median of RNA concentration (Range) ng/μL	RQI score > 7	*A*_260_/*A*_280_ range	*A*_260_/*A*_230_ range
Intact RNA*	279.5 (197.5–340.0)	100%	1.8–1.9	0.6–1.2
Degraded RNA*	320.5 (236.5–369.0)	100%	1.9–2.2	0.6–1.2
RNase treated^#^	112.3 (96.0–128.5)	0%	1.9–1.9	0.3–1.6

* Four biological repeats were tested.

^#^ Two biological repeats were tested.

It has previously been reported that isolated total RNA is preserved after thawing for 24 hours at room temperature (e.g., in the case of freezer breakdown) [21]. We also introduced other inadvisable actions that could potentially introduce RNase to the sample, but these failed to degrade the RNA samples. Therefore, we consider total RNA extracted from muscle samples to be surprisingly stable during storage and handling. Our recommendations for RNA handling and storage are listed in Box 4.

**Box 4**

It is recommended to wear gloves and to have RNase-free working areas, pipettes, barrier tips, and tubes that are restricted to RNA work. It is also suggested to store RNA samples at -80°C and to minimise the number of freeze-thaw cycles [22]. Although RNA samples are very stable, we still recommend handling RNA with care and checking the RNA concentration and quality before cDNA synthesis.

## Step 4: Reverse transcription

### Reagents and protocols

The next step after RNA extraction and quality checks is reverse transcription—the process of synthesising cDNA using the extracted RNA as the template. The reverse transcriptase enzyme uses the RNA template and short-sequence primers to direct the synthesis of the first strand cDNA, which is then used as a template for the qPCR reaction. There are different reverse transcription enzymes and cDNA priming strategies, each with their advantages and disadvantages, and there is no one strategy recommended for all experiments [15].

For all of our experiments, we used Bio-Rad iScriptTM RT Supermix cDNA synthesis kit (Bio-Rad, Hercules, CA) to generate cDNA. The reverse transcriptase enzyme included in this kit degrades the original RNA template after first-strand cDNA synthesis. This can improve the sensitivity of subsequent qPCR analysis, as the RNA template can bind to the newly-synthesised cDNA and stop the primers from binding to the cDNA template during the qPCR reaction. However, the choice of cDNA synthesis kit depends on the individual experiment and laboratory, and other commercially available kits could provide advantages such as greater reverse transcriptation efficiency, greater ease of use, a faster experimental protocol, or reduced cost.

Priming can be performed using oligo-dT primers or random primers, or a mixture of both. Oligo-dT primers are short sequences of deoxy-thymidine nucleotides that are used to produce full-length copies of the mRNA, by binding to the poly(A) tail of mRNA. Random primers can bind throughout the entire length of the RNA, ensuring reverse transcription of all RNA sequences due to their random structure. To achieve unbiased representation of the 5′ and 3′ region of target genes, we used a mixture of oligo-dT and random primers. The cDNA produced was then diluted 5 to 10 times with nuclease free water and stored at -20°C for subsequent analysis. As recommended by major manufacturers of qPCR instruments and reagents, a reaction with all ingredients, including the same amount of template RNA (1 μg) except reverse transcriptase, was carried out as a minus-reverse transcriptase control (˗RT). The ˗RT control contains no cDNA, but contains the same amount of genomic DNA contamination as the cDNA sample; thus it is a useful control for testing if the qPCR primers amplify genomic DNA in the reaction (this result is reported in the next section). Our recommendations for reverse transcription are listed in Box 5.

**Box 5**

It is important to use the same reverse transcription protocol for all samples that will be directly compared, and experimental details need to be reported (e.g., amount of RNA used, type of reverse transcriptase enzyme, type of primers used, as well as reaction volume and temperature; see example in the Materials and methods section). It is also recommended to synthesise the ˗RT control with the samples, which can be used for a primer specificity test (see Step 5 for more information).

## Step 5: qPCR optimisation

### Primer design and testing

During gene expression, DNA is first transcribed into mRNA. In eukaryotes, non-coding regions of the mRNA sequence, known as introns, are removed and the protein-coding regions, known as the exons, are joined to produce the mature mRNA that is translated into protein. This process is called RNA splicing. The human genome contains ~20,000 protein coding genes, which can be processed into more than 80,000 protein-coding mRNA, and the estimated number of proteins synthesised is in the range of 250,000 to 1 million [23]. This suggests the regulation of gene expression is a complicated process. One of the regulatory processes is alternative splicing, in which particular exons of the same gene are joined to produce multiple mRNA, called splice variants, which are then translated into different protein isoforms. We recommend including all splice variants of a target gene unless the user is only interested in one particular splice variant of the target gene. It is also preferable to design primers that bind specifically to cDNA but not genomic DNA, as amplification from genomic DNA could interfere with gene expression analysis. This can be achieved by designing a primer sequence that crosses an exon-exon junction, or by including a large intron between the forward and reverse primer. This is an essential requirement if no genomic DNA elimination step is performed during RNA extraction. In certain circumstances, such as when the target gene sequence does not contain an intron or the primer cannot be located at the exon-exon junction due to the DNA sequence, genomic DNA must be removed from the RNA sample before being converted to cDNA. For example, the gene encoding human heat shock protein family A member 6 (HSPA6) does not contain any introns, and thus genomic DNA must be removed from the RNA sample to prevent the primers from amplifying both genomic DNA and cDNA simultaneously during the qPCR reaction. The advantage of using commercially-available, spin column-based kits to extract RNA is they generally include a genomic DNA elimination step.

**Fig 4:**
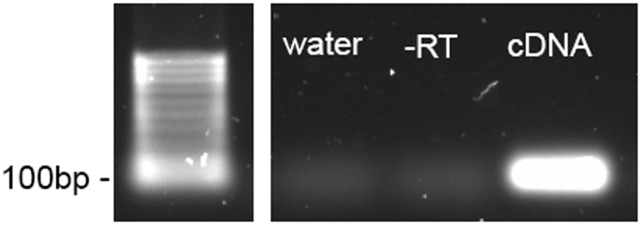
Example of a primer specificity test. DNA fragment products produced from a PCR reaction with the same primers, but using either water (Lane 2), -RT (cDNA synthesis reaction containing RNA but no cDNA, Lane 3), or cDNA (Lane 4) as template, were separated on a 2% agarose gel. Lane 1 is the 100 bp DNA ladder. A single sharp DNA band of the expected size, which is the final PCR amplicons, is present only in the reaction with cDNA.

To test if primers are specific to cDNA, one approach is to perform qPCR reactions using cDNA, a ˗RT control, or water as template. The final PCR amplicons can then be separated using electrophoresis with a 2% agarose gel. A single sharp DNA band of expected size should be present only in the reaction with cDNA if the primers are only binding to cDNA (Fig 4).

**Fig 5:**
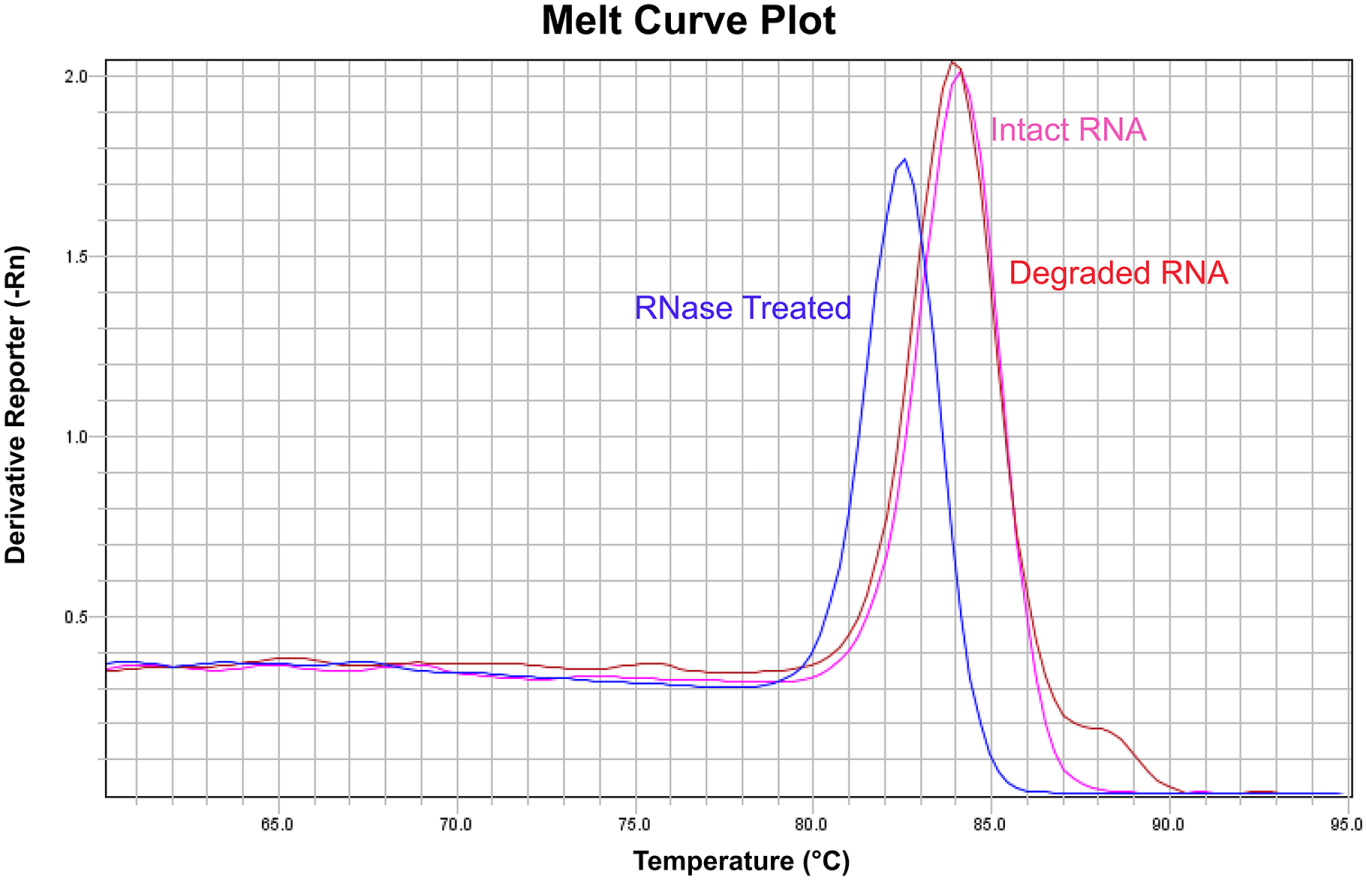
An example of the melting curve analysis of a single RNA sample under three different storage/treatment conditions. The qPCR reaction using cDNA synthesised from Intact (pink) and Degraded RNA (red) sample show the same melting curve, indicating that the same PCR amplicon is produced. However, a different melting curve is observed when using cDNA synthesised from RNase Treated RNA sample (blue), which shows a different PCR amplicon is produced during the qPCR reaction. Our recommendations for qPCR optimisation are listed in Box 6.

Primer specificity can also be checked by melting curve analysis. Melting curve analysis is an assessment of the dissociation characteristics of double-stranded DNA (the product from the qPCR reaction) during heating, and can be used in qPCR reactions with intercalating dyes, such as SYBR Green. SYBR Green only fluoresces when it is bound to double-stranded DNA, but not in the presence of single-stranded DNA. At the end of a qPCR run, the thermal cycler is programmed to increase the temperature gradually from 60°C to 95°C (0.05°C·s^-1^) and to measure the amount of fluorescence. The double-stranded PCR amplicon begins to denature to single-stranded DNA, resulting in decreased fluorescence. The temperature at which the base-base hydrogen bonding between two DNA strands is broken depends on their length, guanine-cytosine content, and their complementarity; thus, a unique melting curve of the changing rate of fluorescence (-Rn) versus temperature will be produced for each specific double-stranded DNA fragment (Fig 5). If more than one DNA fragment is produced during the qPCR reaction, using cDNA and genomic DNA as the template respectively, more than one melting curve will be detected. We highly recommend including melting curve analysis with SYBR Green-based qPCR analysis, and ensuring a single specific product is produced in all reactions amplifying the same target gene. This can be easily done by adding the melting curve temperature program at the end of a qPCR run, which is a function available in most qPCR instruments.

**Box 6**

It is recommended to design qPCR primers that are specific to the target gene and only amplify cDNA (e.g., target all intended isoforms, and span a junction between exons), and the primer specificity needs to be tested as part of the qPCR validation process [1]. We recommend including a melting curve analysis to test primer specificity, as it requires no extra step. According to the MIQE guidelines, authors are required to provide sufficient information on the qPCR target and primers, such as gene symbol, sequence accession number, and amplicon length [1] (see example in Experiment 4).

### Optimising qPCR performance

In a qPCR reaction, the quantification cycle (C_q_) value is defined as the number of cycles required for the fluorescent signal to exceed the background fluorescence (also referred to as threshold cycle (C_t_), crossing point (C_p_), or take-off point (TOP) in previous publications). The qPCR software programs can set the threshold automatically after determination of the baseline fluorescence from cycle 3 to 15 across the entire reaction plate, which is known as the baseline value. By default, the QuantStudio Real-Time PCR software program (Applied Biosystems, Foster City, CA), which we use in our laboratory, sets this threshold at ten standard deviations above the mean baseline fluorescence. However, both the baseline and threshold can be adjusted manually.

**Fig 6:**
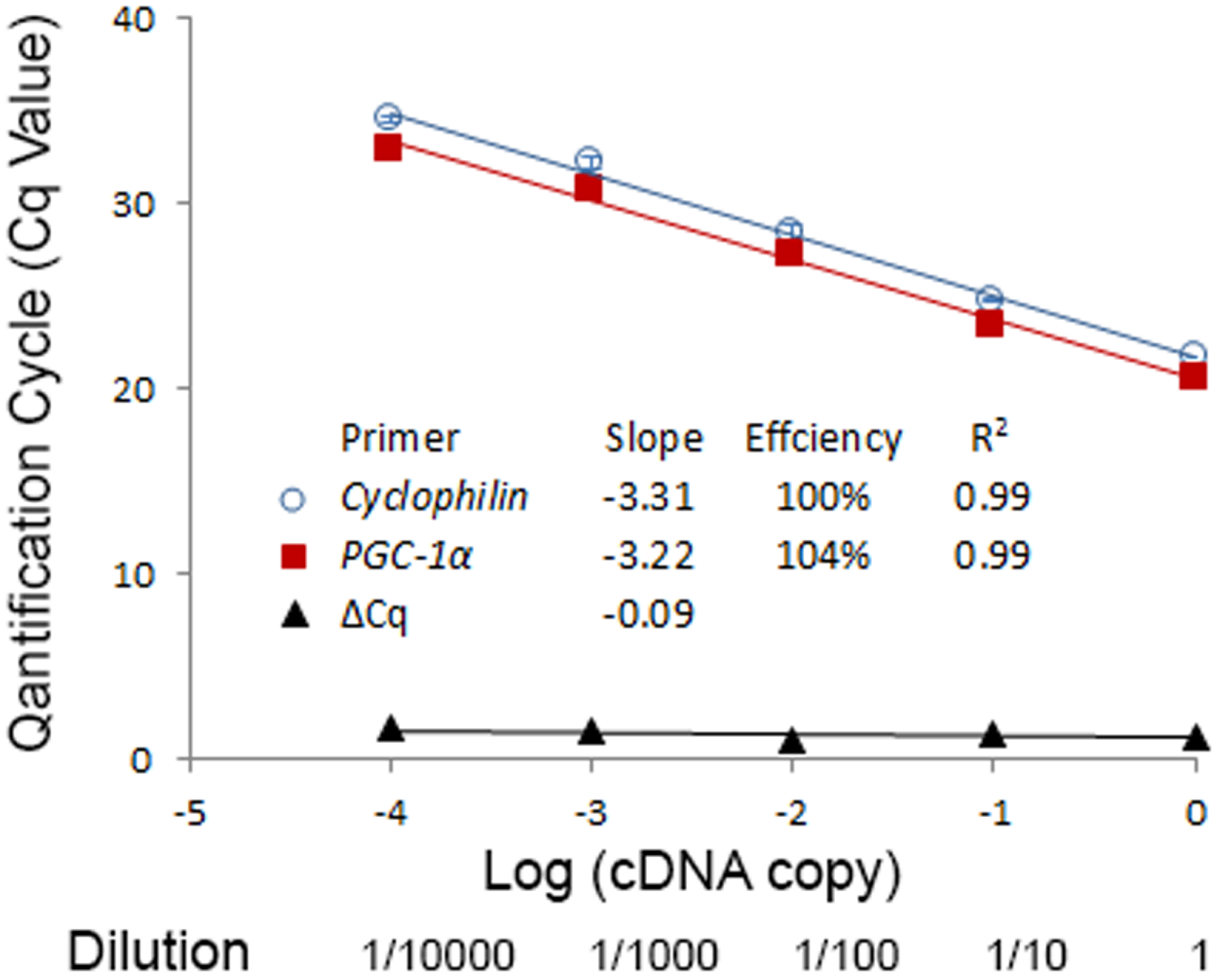
An example of an amplification efficiency test using *Cyclophilin* (see Table 4 for details) and *PPARG coactivator 1 alpha* (*PGC-1α*, see Experiment 4 for details) primers. A standard curve was generated using a 10-fold dilution of cDNA as template for qPCR reactions. The resulting C_q_ values are plotted against the Log of the cDNA input. The efficiency, as well as the R^2^ value, are within the acceptable range. The efficiencies of *Cyclophilin* and *PGC-1α* are approximately equal, as the absolute value of the slope of ΔC_q_ against the Log of the cDNA input is < 0.1.

To obtain high amplification efficiency (an increase in number of PCR amplicons per cycle), both the primer and cDNA concentration need to be optimised for different target genes. The recommended amplification efficiency is between 93% and 105% (the slope of the C_q_ against the Log of the cDNA input in a standard curve is between -3.2 and -3.5 and the R^2^ value is above 0.98; see examples in Fig 6) [15].

The choice of cDNA concentration for the final qPCR reaction will depend on the qPCR kit of choice, the primers used, as well the expression level of the target gene. In our laboratory, we first dilute cDNA ten times with water before using in any qPCR reactions. When testing a new set of primers, a standard curve should be generated using a series of diluted cDNA samples as template (Fig 6, using *Cyclophilin* and *PGC-1α* as an example,). A “no template” control reaction should be set up using only water (template free control, TFC). qPCR amplification efficiency can then be calculated from the slope of the graph of C_q_ values plotted against the Log of the cDNA input (Efficiency = (10^−1/slope^– 1) × 100). New sets of primers should be designed and tested if the amplification efficiency is not within the recommended range.

For all experiments described in this paper, we used an initial primer concentration of 300 nM. It is recommended to choose primer and cDNA concentrations within the linear dynamic range for qPCR, which results in a C_q_ of between 20 to 30 [15]. However, in reactions with a high C_q_ value (>30, depending on the expression level of target gene and the qPCR protocol), it is necessary to run qPCR reactions with different primer concentrations, and to use the concentration that gives the lowest C_q_ value (indicating the reaction was performed under the most efficient conditions). Different qPCR reaction kits may recommend a different primer concentration. Our recommendations for optimising qPCR performance are listed in Box 7.

**Box 7**

It is suggested to keep the machine’s default setting as the threshold, but to always check if it has been set in the region of exponential amplification across all amplification plots, and that all plots are parallel and above the background noise of the baseline [15]. Each qPCR reaction should be optimised to achieve a preferred C_q_ value (20 to 30) and amplification efficiency (93%–105%). For some target genes, it is difficult to obtain primers with the desired amplification efficiency even after testing multiple primer sets. We recommend using primers with an amplification efficiency closest to the desired range of between 93% and 105% (see primers for *ACTB* and *GAPDH* as examples in Experiment 4).

## Step 6: qPCR assay

### Reagent and protocol

The two most commonly used qPCR chemistries for gene expression analysis are hydrolysis probe and DNA binding dyes. Hydrolysis probe-based detection requires a pair of unlabelled primers, and a Taqman^®^ probe (short sequence-specific DNA fragment that can bind to a target gene sequence) with a fluorescent dye label on the 5′  end (the end of the DNA strand that has a fifth carbon in the deoxyribose sugar) and a quencher on the 3′ end (the end of the DNA strand that has a third carbon in the deoxyribose sugar) to quench the fluorescent dye. During the PCR cycle, both the primers and the probe will anneal to the target sequence. When new DNA strands are synthesised by the enzyme (*Taq* DNA polymerase), the enzyme cleaves the probe and separates the dye from the quencher. During each cycle, more dye molecules are released from the probe, and this results in an increase in fluorescence intensity proportional to the amount of new DNA strands synthesised. Hydrolysis probe chemistry is specific to target genes; thus it is more sensitive and reproducible than DNA binding dyes when measuring lowly-expressed genes [24].

SYBR Green is a popular DNA binding dye used in qPCR [25], which only fluoresces when bound to double-stranded DNA and not in the presence of single-stranded DNA. The cost of employing SYBR Green as a qPCR detection method is much lower than hydrolysis probes, and it has been suggested to be the most cost-effective chemistry for initial investigations and primer optimisation steps [15]. In our experiments, only SYBR Green chemistry was used and is subsequently discussed.

qPCR should be performed with several technical repeats. The variation between technical repeats can depend on the qPCR instrument, the detection method, the reaction volume, and the liquid dispensing method. To improve reliability, qPCR instruments should be calibrated and tested regularly according to the manufacturer’s instructions. One of the most critical aspects is the dispensing method, as it is important to achieve accurate dispensing of reaction components. It is recommended to perform the reaction at least in triplicate and to avoid pipetting less than 3 μL of reagents if the reaction is prepared manually [15]. The average C_q_ value from technical repeats is used for calculations. However, when using an automated system, fewer repeats are required. For example, we conducted qPCR assay on samples from previously described experiments (Experiments 1, 2 and 3) in duplicate using an automated pipetting system (detailed are described below), and we observed that 100% of the samples had a standard deviation (SD) less than 0.4 for C_q_, while 96% of the samples had a SD less than 0.3 for C_q_. This supports the use of two repeats with an automated pipetting system. Our recommendations for running qPCR assay are listed in Box 8.

**Box 8**

Complete reaction conditions, including reaction volume, the concentration of all components, and thermocycling parameters and instruments, are essential to report [1]. The number of technical repeats depends on the reaction volume and dispensing methods. We recommend conducting experiments in duplicate if an automatic dispensing system is used, and in triplicate if pipetting manually.

## Step 7: Data analysis

### A. Data normalisation—Comparative C_q_ method

The importance of normalisation of qPCR data has been emphasised repeatedly [1, 26]. Data normalisation is a critical step in the qPCR workflow, as it corrects for variations in multiple steps, including RNA purification, RNA concentration assessment, as well as reverse transcription and amplification efficiency. Normalisation with stably expressed reference genes as internal controls, known as the comparative C_q_ or the ΔΔC_q_ method, is the most common method for the normalisation of mRNA data. However, this technique requires appropriate validation to make sure it is performed correctly [27]. For the comparative C_q_ method to be valid, it is important to make sure the reference genes and target genes have a similar amplification efficiency, as a valid comparative C_q_ method is based on an additional assumption of similar amplification efficiency [28]. A standard curve can be plotted for the ΔC_q_ (the difference between reference and target gene against the log of cDNA input), and the absolute value of the slope should be <0.1 [29]. See an example of ΔC_q_ between *Cyclophilin* and *PGC-1α* in Fig 6. If it is not possible to obtain reference genes with similar amplification efficiency as the target, it is suggested to use the Pfaffl method for calculation, in which the calculation is adjusted by the differences in the amplification efficiency of the target and reference genes [30, 31].

The comparative C_q_ method normalises the C_q_ value of a target gene to internal reference genes before comparisons are made between samples. First, the difference between C_q_ values (ΔC_q_) of the target gene and the geometric mean of multiple reference genes is calculated for each sample, and then the difference in the ΔC_q_ (ΔΔC_q_) is calculated between two samples (e.g., control and treatment, or pre and post treatment). The fold-change in expression of the two samples is calculated as 2^-ΔΔCq^, where 2 derives from 1 + efficiency and efficiency is assumed to be 1 (i.e., 100% efficiency) [28]. Our recommendation for using comparative C_q_ method is listed in Box 9.

**Box 9**

It is important to ensure that the reference genes and target genes have a similar amplification efficiency when using the comparative C_q_ method; otherwise the Pfaffl method should be considered.

### B. Choice of reference genes

Several traditional reference genes have been widely used in the qPCR analysis. A review article has reported that 33% and 32% of the expression analysis from 6 high-impact journals used *glyceraldehyde-3-phosphate dehydrogenase* (*GAPDH*) and *actin beta* (*ACTB*) as reference genes respectively for papers published in 1999 [32]. However, the same review pointed out that the expression of both *GAPDH* and *ACTB* varies considerably under different experimental settings in a range of tissues [32]. *GAPDH* was originally identified as an intermediate in glycolysis pathway and expected to be stably present in all cells; thus it was selected as a reference gene. However, other activities of *GAPDH*, including functions in endocytosis, translational control, and DNA replication [33], were not recognised until later [32].

Various reference genes have been used in different exercise studies. Mahoney et al. -[34] reported that *β-2-microglobulin* (*B2M*) and *ACTB* were the most stable reference genes following 300 eccentric contractions, whereas *B2M* and *GAPDH* were the most stable following 75 min of high-intensity intermittent cycling. In another study, muscle biopsies were taken before, immediately after, and 4 h following 30 min of treadmill running at 70% of VO_2max_, and RNA was extracted from 40 single fibres. *GAPDH* was found to be stably expressed in all samples [35]. Thus, when using reference genes as internal controls for an exercise study, the stability of each reference gene needs to be evaluated carefully, and there is no ‘one-size-fits-all’ gene that can be used in all studies and with all exercise protocols.

In order to reduce the variability of internal control, it is recommended to use multiple genes for normalisation [36, 37]. Using samples obtained from neuroblastoma cell lines, Vandesompele and his colleagues showed that normalisation using a single reference gene led to differences of 3.0-fold in 25%, and 6.4-fold in 10%, of the cases analysed [36]. The evaluation of reference genes can be achieved by running a statistical analysis on the C_q_ value or using available software. Several software programs are available for reference gene evaluation using different analytical approaches, such as BestKeeper [38], NormFinder [39], and GeNorm [36].

In our laboratory, we have six commonly-used reference genes, *ACTB*, *TATA-box binding protein* (*TBP*), *Cyclophilin*, *GAPDH*, *B2M*, and *18S rRNA* (Table 4). There are a few reasons why these candidate reference genes were chosen. First of all, these six genes are potentially stably-expressed reference genes, and are widely used in the qPCR analysis of skeletal muscle samples obtained in human exercise studies [34, 35, 40]. Second, they belong to different functional classes; thus it is unlikely they are co-regulated. However, using comparative C_q_ method, it is difficult to qualify small differences in gene expression (i.e., less than 2-fold) unless multiple stably-expressed reference genes are used for normalisation [36, 41].

**Table 4:** Function of common reference genes used in exercise studies.

Gene	Accession no.	Function (NCBI Reference sequence database [42])
*ACTB (actin beta)*	NM_001101.3	This gene encodes one of six different actin proteins, which is a major constituent of the contractile apparatus and one of the two nonmuscle cytoskeletal actins.
*TBP (TATA-box binding protein)*	NM_003194.4	This gene encodes a general transcription factor that binds specifically to a DNA sequence called the TATA box, and helps position RNA polymerase II over the transcription start site of the gene.
*Cyclophilin (PPIA*, *peptidyl-prolyl cis-trans isomerase A)*	NM_021130.4	This gene encodes a protein that catalyses the cis-trans isomerization of proline imidic peptide bonds in oligopeptides and accelerates the folding of proteins.
*GAPDH (glyceraldehyde-3-phosphate dehydrogenase)*	NM_001289746.1	This gene encodes a key enzyme in the glycolytic pathway, which catalyses the reversible oxidative phosphorylation of glyceraldehyde-3-phosphate in the presence of inorganic phosphate and nicotinamide adenine dinucleotide (NAD).
*B2M (β-2-microglobulin)*	NM_004048.2	This gene encodes a serum protein in association with the major histocompatibility complex (MHC) class I heavy chain on the surface of nearly all nucleated cells.
*18S rRNA (RNA*, *18S ribosomal)*	NR_003286.2	This gene represents the portion of one rDNA repeat which encodes a 18S rRNA.

#### Experiment 4

**Fig 7:**
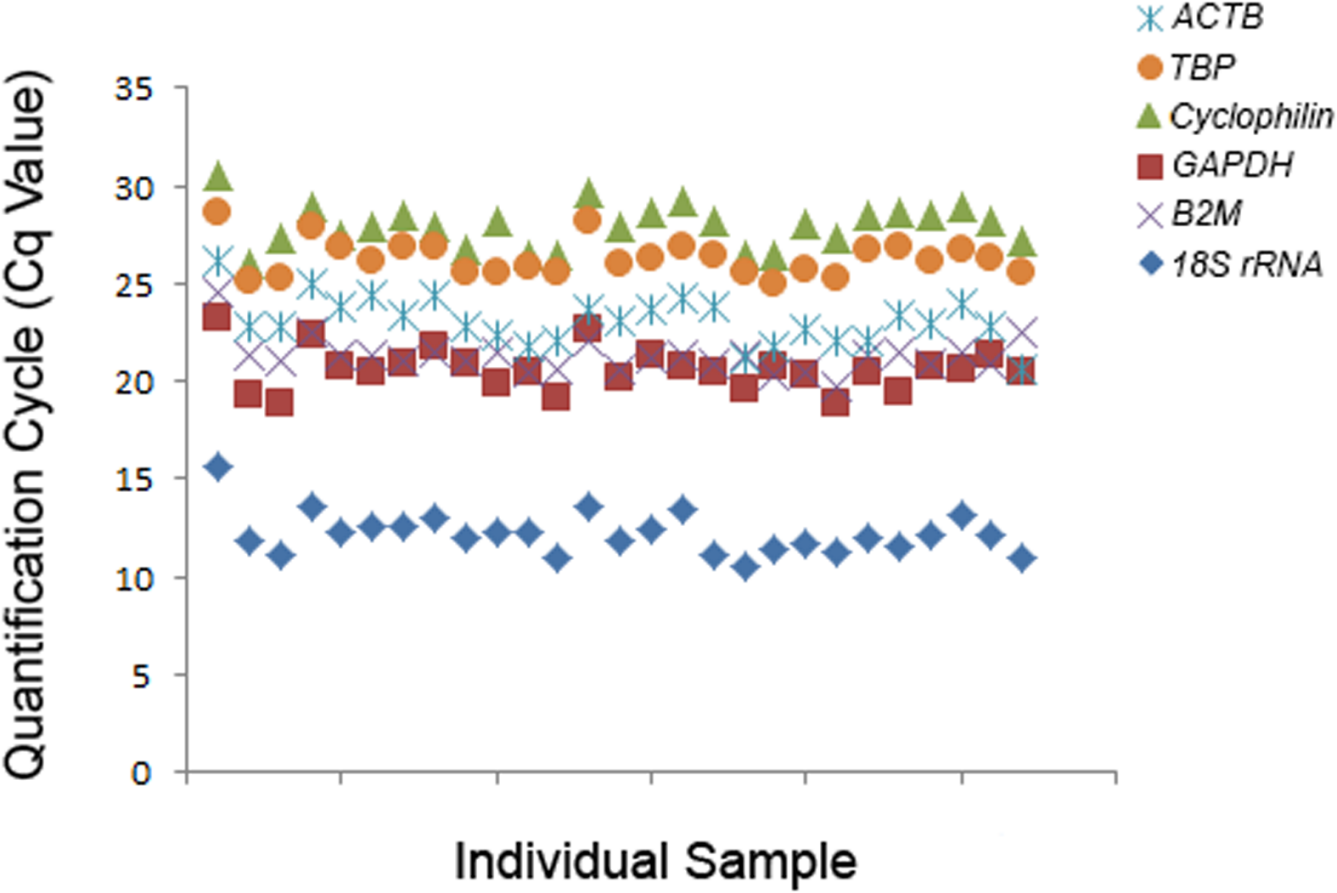
Expression of six commonly-used reference genes in exercise studies. C_q_ values of individual reactions using *ACTB*, *TBP*, *Cyclophilin*, *GAPDH*, *B2M*, and *18S rRNA* primers are presented. All samples are from an exercise study (n = 9 participants × 3 time points = 27 samples).

In our human exercise study (see Materials and methods), muscle samples were taken from 9 participants at rest (Baseline), and then immediately post (0 h) and 3 h post (3 h) the final training session of a 4-week training intervention. All muscle samples were snap-frozen immediately after the muscle biopsy, and the RNA for all samples (n = 27) was extracted using RNeasy Plus Universal Mini Kit with a modified protocol (using 2-propanol); for all samples we obtained an *A*_260_/*A*_280_ ratio greater than 1.9 and an RQI score greater than 7 (using an Experion automated electrophoresis system). We then performed the reverse transcription to convert RNA into cDNA in a single run, before conducting qPCR analysis. To find stably expressed reference genes across all samples at all time points, we tested six reference genes (*ACTB*, *TBP*, *Cyclophilin*, *GAPDH*, *B2M*, and *18S* rRNA; Table 5 and Fig 7). We then used RefFinder to evaluate the stability of these genes. RefFinder is a web-based tool, which is able to run four well-established algorithms simultaneously (GeNorm [36], BestKeeper [38], NormFinder [39] and comparative delta-CT [43]), assign an appropriate weight to each individual gene, and calculate the geometric mean of their weights for the overall final ranking [44]. Based on the recommended comprehensive ranking from RefFlinder, our candidate genes were ranked from most to least stable as *B2M*, *TBP*, *18S rRNA*, *ACTB*, *GAPDH* and *Cyclophilin* (Table 5).

**Table 5:** Evaluation of reference genes using RefFinder.

Ranking Order (Most to least stable)						
Method	1	2	3	4	5	6
Delta CT	*TBP*	*B2M*	*18S rRNA*	*ACTB*	*GAPDH*	*Cyclophilin*
BestKeeper	*B2M*	*TBP*	*18S rRNA*	*ACTB*	*GAPDH*	*Cyclophilin*
NormFinder	*TBP*	*B2M*	*18S rRNA*	*ACTB*	*GAPDH*	*Cyclophilin*
Genorm	*B2M / 18S rRNA*		*TBP*	*ACTB*	*GAPDH*	*Cyclophilin*
**Recommended comprehensive ranking**	*B2M*	*TBP*	*18S rRNA*	*ACTB*	*GAPDH*	*Cyclophilin*

To illustrate how one might go about choosing reference genes, we choose *PPARG coactivator 1 alpha* (*PGC-1α*) as an example for gene expression analysis. *PGC-1α* is a transcriptional coactivator that is enriched in skeletal muscle. It has been shown that exercise is able to increase *PGC-1α* mRNA content in humans [45]. The amplification efficiency of all six candidate reference genes was similar to our target gene, *PGC-1α* (Table 6). We used the geometric mean of top three ranked genes by RefFinder (*TBP*, *B2M*, and *18S rRNA*) for subsequent data normalisation. Our recommendations for choosing reference genes are listed in Box 10.

**Table 6:** Primer sequences and amplicon details.

Gene	Accession no.	Primers (Forward and Reverse)	Amplicon size (bp)	Start position (bp)	Efficiency (%)	Source
*TBP (TATA-box binding protein)*	NM_003194.4	F: CAGTGACCCAGCAGCATCACT R: AGGCCAAGCCCTGAGCGTAA	205	121	99	[46]
*Cyclophilin (PPIA*, *peptidyl-prolyl cis-trans isomerase A)*	NM_021130.4	F: GTCAACCCCACCGTGTTCTTC R: TTTCTGCTGTCTTTGGGACCTTG	100	93	100	[47]
*B2M (β-2-microglobulin)*	NM_004048.2	F: TGCTGTCTCCATGTTTGATGTATCT R: TCTCTGCTCCCCACCTCTAAGT	86	589	98	[36]
*ACTB (actin beta)*	NM_001101.3	F: GAGCACAGAGCCTCGCCTTT R: TCATCATCCATGGTGAGCTGGC	70	26	107	Designed by authors
*18S rRNA (RNA*, *18S ribosomal 5)*	NR_003286.2	F: CTTAGAGGGACAAGTGGCG R: GGACATCTAAGGGCATCACA	71	1443	99	[48]
*GAPDH (glyceraldehyde-3-phosphate dehydrogenase)*	NM_001289746.1	**F: AATCCCATCACCATCTTCCA** **R: TGGACTCCACGACGTACTCA**	82	388	106	[49]
*PGC-1α (PPARG coactivator 1 alpha*)	NM_013261.3	F: CAGCCTCTTTGCCCAGATCTT R: TCACTGCACCACTTGAGTCCAC	101	199	104	[50]

**Box 10**

It is recommended to test multiple reference genes [36, 37], and the stability of each gene should be assessed before choosing appropriate reference genes for a particular study. We recommend using RefFinder to assess the stability of reference genes, as it runs four well-established algorithms simultaneously. Other than the stability of the reference gene, the amplification efficiency of the reference genes should be similar to the target genes.

### C. Normalising gene expression via cDNA quantification

Finding stable reference genes is a challenge when performing qPCR, and researchers have been seeking alternative methods such as quantifying cDNA. Quant-iT^™^ OliGreen ssDNA reagent is a fluorescent nucleic acid dye for quantifying cDNA, and it has been used in many published papers including studies investigating gene expression in human skeletal muscle in response to exercise [51–53]. A potential problem with using OliGreen dye to quantify cDNA content is that the dye is also sensitive to RNA, as stated in the user manual “*the OliGreen reagent does exhibit fluorescence enhancement when bound to RNA*” [54].

#### Experiment 5

**Fig 8:**
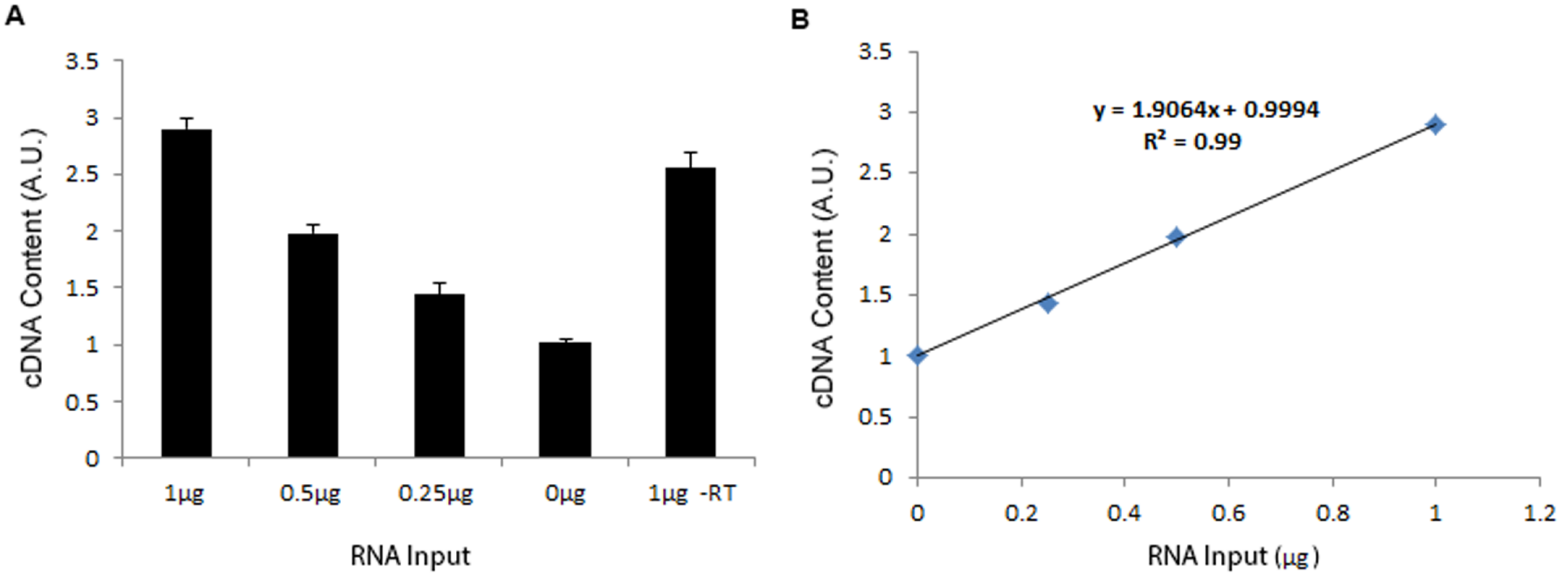
A: Determination of cDNA amount in reactions with different RNA input. Different amounts of RNA were used to synthesise cDNA (n = 4 for each RNA input), and the relative amount of cDNA in each reaction was measured using OliGreen dye. Values are presented as mean ± SD. B: Correlation between RNA input and average relative amount of cDNA measured.

To test the specificity and validity of using OliGreen dye to qualify cDNA content, we synthesised cDNA from four different amounts (0, 0.25, 0.5, and 1 μg) of RNA obtained from Experiment 1 (‘Good Practice”, n = 4 for each RNA input). We also loaded 1 μg RNA in the–RT control reaction, which contained no cDNA (n = 4). We used iScript^™^ Reverse Transcription Supermix (Bio-Rad) for cDNA synthesis. The enzyme reverse transcriptase in this kit has RNase H^+^ activity that degrades the RNA strand in RNA-DNA hybrids after cDNA synthesis. The cDNA content in each sample was then measured using OliGreen dye (Fig 8A). Consistent with previous research [51], cDNA samples synthesised from different amounts of RNA showed a strong positive correlation for the measured cDNA concentration versus RNA input (r = 0.9947, *P*< 0.0001) (Fig 8B). However, the -RT control reaction, which contained only 1 μg RNA but no cDNA, showed a higher reading than cDNA synthesised from 0.5 μg RNA. This result confirmed that the OliGreen dye is not specifically measuring ssDNA, but measures RNA as well. This could cause a false high cDNA content in the assay if RNA is not degraded properly. Our recommendations for normalising gene expression via cDNA quantification are listed in Box 11.

**Box 11**

In order to obtain an accurate and specific measurement of cDNA from the Oligreen dye, RNA in the RNA-DNA hybrids needs to be degraded before measurement. This can be achieved by using a reverse transcriptase enzyme with RNase H^+^ activity (Fig 8), or including an RNase degradation step after cDNA synthesis [51].

### D. Effect of normalisation methods on the results

#### Experiment 6

**Fig 9:**
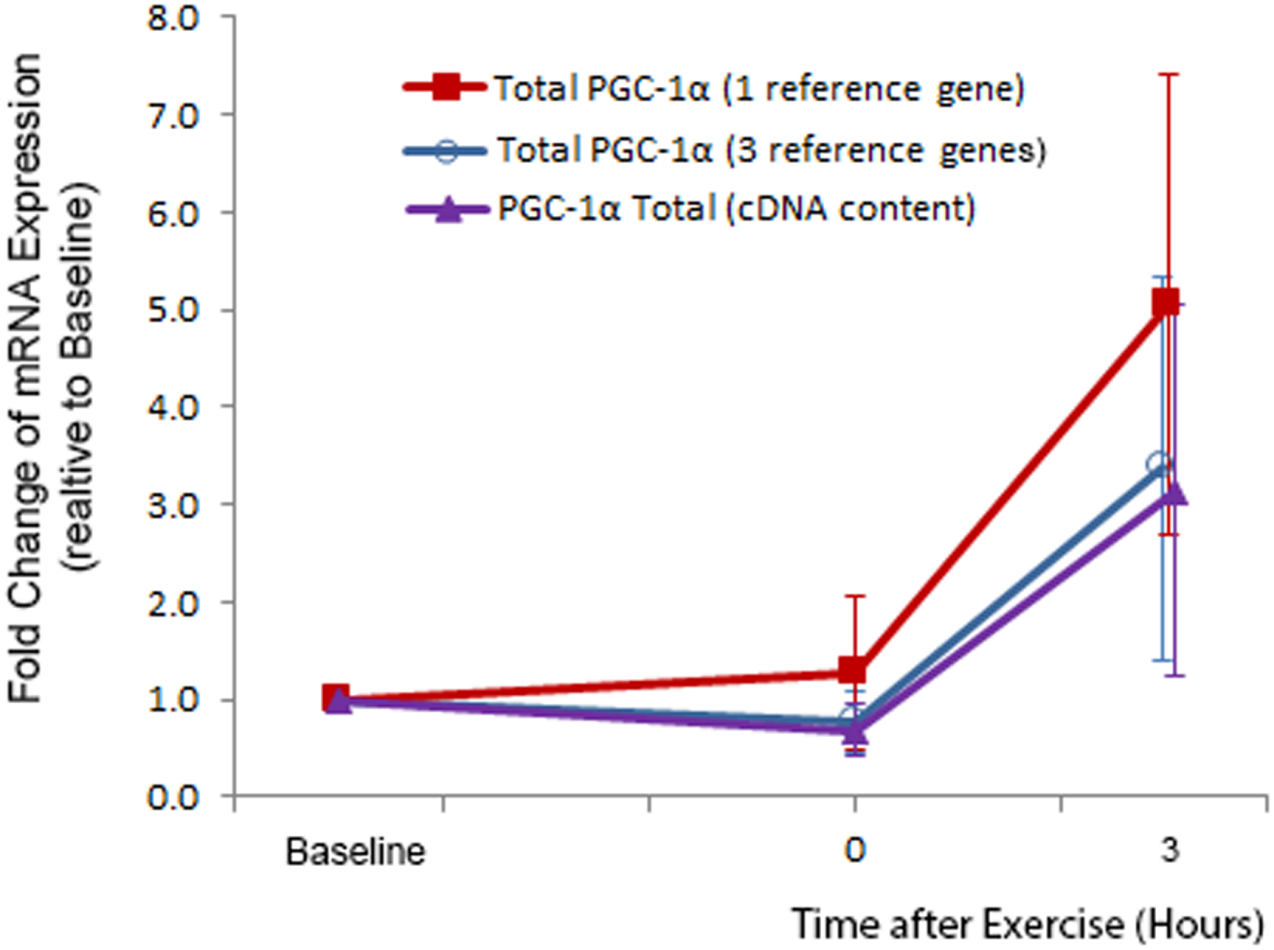
Expression of *PGC-1α* mRNA in an exercise study with 9 participants. Muscle samples were taken at rest (Baseline, Week 0) and immediately post-exercise (0 h), and 3 h post-exercise. Data were analysed using 3 different normalisation methods. Values are fold change ± SD.

To investigate how normalisation might alter the outcome, we measured the exercise-induced expression of *PGC-1α* mRNA in the samples from a human exercise study (as described in Experiment 4) and analysed the same set of data in three ways. We performed normalisation using three of the most stable reference genes (*TBP*, *B2M*, and *18S rRNA*), based on the reference gene evaluation (Table 5). In comparison, we also used a single reference gene, *Cyclophilin*, which was the lowest ranked reference gene. Lastly, we analysed the data using the cDNA content measured by Quant-iT^™^ OliGreen ssDNA Reagent. We saw a significant difference in gene expression at 3 hours after exercise using all three normalisation options (P < 0.01, Fig 9). These exercise-induced fold changes in *PGC-1α* expression are consistent with the existing literature [40, 45].

There were no significant differences between the fold changes in *PGC-1α* mRNA content when using different methods of normalisation; however, the fold changes were more similar when using three reference genes and cDNA content for normalisation, rather than using one reference gene. The increase of *PGC-1α* was 3.4 ± 2.0 fold when three reference genes were used for normalisation. When a single reference gene (*Cyclophilin*) was used for normalisation, the increase in gene expression was 5.1 ± 2.4 fold (P = 0.08 compared to 3 reference genes). When cDNA content was used for normalisation, the increase of gene expression was 3.2 ± 1.9 fold (*P* = 0.51 compared to 3 reference genes, *P* = 0.09 compared to 1 reference gene). In certain experimental settings, especially when examining small changes in mRNA level, these different results could lead to different conclusions. This may also help to explain the inter-study variability for exercise-induced changes in mRNA content. As previously suggested, use of a single reference gene is considered ‘not acceptable’ unless its stability has been clearly demonstrated in the same study [1]. Our recommendations for normalising gene expression via reference genes are listed in Box 12.

**Box 12**

We recommend testing four or more candidate reference genes for each study, and selecting the ones that are stably expressed for data normalisation. In our research laboratory, we chose to use two to three most stable reference genes based on evaluation software for an individual study [45, 55].

## Conclusions

Examining gene expression responses to exercise training by qPCR provides a deeper understanding of the molecular mechanisms underpinning physiological changes observed in exercise studies. However, there is considerable variation in how different laboratories perform qPCR experiments, which can make it difficult to compare results between studies. To highlight the importance of various steps in the qPCR workflow, we conducted several experiments to show how methodological variations may affect the final gene expression result. We also presented qPCR results from an exercise study, where nine participants performed a single session of high-intensity interval exercise.

Fig 2 provides a workflow to show researchers the steps from processing muscle samples from a biopsy to qPCR analysis. We also discussed the effects of common methodological variations and provided recommendations at each step. Together with a detailed checklist of the information required when preparing a report that includes qPCR analysis in the MIQE guidelines [1], the information in this paper will assist readers to design and perform qPCR analysis in muscle samples from an exercise study, and to obtain data that are more reliable.

## Materials and methods

### Participants

All studies had been approved by the Victoria University Human Research Ethics Committee. All participants gave written informed consent to participate in these studies. The muscle samples used for Experiments 1, 2, 3 and 5 were obtained from four recreationally-active men [age: 21 (2) y; height: 173.4 (5.1) cm; mass: 71.9 (5.0) kg; ${\dot{\text{V}}\text{O}}_{\text{2}}{}_{\text{peak}}$: 46.4 (6.1) mL·min^-1^·kg^-1^; mean (SD)]. The muscle samples used for Experiments 4 and 6 were obtained from nine recreationally-active men [age: 23 (4) y; height: 180.5 (8.4) cm; mass: 81.6 (13.1) kg; ${\dot{\text{V}}\text{O}}_{\text{2}}{}_{\text{peak}}$: 49.2 (6.9) mL·min^-1^·kg^-1^; mean (SD)].

### Experimental design for Experiments 4 and 6

Nine recreationally-active men underwent a resting muscle biopsy (Week 0) before undertaking four weeks of high-intensity interval training (HIIT) as part of a related study [56]. In week 4, two muscle biopsies were performed between 06.30 and 08.00 following an overnight fast after the final session of the HIIT intervention. Exercise consisted of seven 2-min intervals performed on an electromagnetically-braked cycle ergometer (Velotron, Racer-Mate, Seattle, WA), with each interval separated by 1 min of passive recovery (2:1 work:rest). The exercise intensity was the power at the lactate threshold (LT), plus 40% of the difference between the power at the LT and the peak aerobic power (W_peak_) [(LT) + 40% (W_peak_ − LT)]. The LT and W_peak_ were determined from a graded-exercise test (GXT) conducted before the HIIT intervention (starting at 90 to 150 W, with 30 W increments every 4.5 min). A familiarisation trial of the GXT was performed on a separate day prior to baseline testing. A standardised 5-min steady-state warm-up at 75 W was completed before the GXT.

### Sample acquisition and handling

During supine rest, and after the injection of a local anaesthetic (1% Xylocaine) into the skin and fascia, a small incision was made in the vastus lateralis of the non-dominant leg and a muscle sample was taken (~150 to 300 mg wet weight) using a Bergström biopsy needle with manual suction applied [57]. Muscle samples were then processed, cleaned of excess blood, fat, and connective tissue using a pair of fine forceps and blotting paper, and then immediately frozen in liquid nitrogen and stored at -80°C. For Experiments 1, 2, 3 and 5, muscle samples were taken from the non-dominant leg at rest. For Experiments 4 and 6, muscle samples were taken at rest in Week 0, and immediately post-exercise (0 h), and 3 h post-exercise in Week 4. Muscle samples were chipped in a -20°C chamber to obtain the desired size, and then returned to -80°C freezer with no extra freeze-thaw cycles until subsequent RNA extraction.

### RNA extraction

To compare the various extraction methods, RNA extraction was performed using TRIzol Reagent (Invitrogen, Thermo Fisher Scientific, Waltham, USA) and RNeasy Plus Universal Mini Kit (Qiagen, Valencia, USA) in Experiment 2. For all other experiments, RNA samples were processed using RNeasy Plus Universal Mini Kit (with the modification described below).

For the TRIzol extraction, total RNA was extracted from approximately 10 to 20 mg of frozen muscle. Samples were homogenised using a TissueLyser II (Qiagen, Valencia, USA) for 2 x 2 mins at 30 Hz with TRIzol^®^ Reagent. The homogenate was centrifuged for 15 min at 12,000 g, and the RNA containing supernatant was removed. The homogenate was combined with chloroform (Sigma-Aldrich, St Louis, USA), and total RNA was then extracted in accordance with the manufacturer’s instructions.

For the RNeasy Plus Universal Mini Kit extraction, total RNA was isolated from approximately 10 to 20 mg of frozen muscle following homogenisation in QIAzol lysis reagent using a TissueLyser II. In order to increase RNA yield, kit instructions were modified by replacing ethanol with 2-propanol to precipitate the RNA. A genomic DNA elimination step was included in the kit to remove genomic DNA from the total RNA.

### RNA assessment

Total RNA concentration was measured using a BioPhotometer (Eppendorf AG, Hamburg, Germany). The purity of each sample was also assessed from the *A*_260_/*A*_230_ absorption ratio. The RNA quality of all samples was measured using a Bio-Rad Experion microfluidic gel electrophoresis system with RNA StdSens Chips (Bio-Rad, Hercules, CA), and determined from the RNA quality indicator (RQI). According to manufacturer’s instructions, samples with a RQI greater than seven were considered as acceptable for qPCR analysis. RNA samples from all experiments, except one sample that was extracted using 2-propanol, obtained a RQI greater than seven (Tables 1 and 2). RNA was stored at -80°C without freeze-thawing until reverse-transcription was performed.

### Reverse transcription

First strand cDNA was then generated from 1 μg of template RNA using a Thermocycler (Bio-Rad, Hercules, CA) and Bio-Rad iScript^™^ RT Supermix (Bio-Rad, Hercules, CA) in a 20 μL reaction, according to the manufacturer’s instructions. Priming was performed at 25°C for 5 min and reverse transcription for 30 min at 42°C using a mixture of oligo-dT primers and random primers. The cDNA was then stored at -20°C for subsequent analysis. The cDNA concentration of a subset of the samples, including -RT, was quantified using Quant-iT^™^ OliGreen ssDNA Reagent (Applied Biosystems, Foster City, CA) according to the manufacturer’s instructions.

### qPCR optimisation and assay

qPCR was performed with a QuantStudio 7 Flex (Applied Biosystems, Foster City, CA). Primers were either adapted from existing literature or designed using Primer-BLAST (http://www.ncbi.nlm.nih.gov/tools/primer-blast/) to include all splice variants, and were purchased from Sigma-Aldrich. Detailed information and instructions for using Primer-BLAST have been discussed previously [58]. Primer specificity was confirmed from the separation of amplification products by 2% agarose gel electrophoresis (Fig 4) and melting curve analysis (Fig 5). The qPCR reaction (5 μL) contained 300 nM of each forward and reverse primer (Table 6) and 2X SsoAdvanced Universal SYBR Green Supermix (Bio-Rad, Hercules, CA), except when amplifying TBP, where 900 nM of each primer was used. The amount of cDNA used in each qPCR reaction was: 0.2 μL for *TBP* and *ACTB*, 0.1 μL for *PGC-1α*, and 0.006 μL for *B2M*, *18S rRNA* and *GAPDH*; these were pre-determined by testing serial dilutions of cDNA samples (see Fig 6 for example). The standard thermocycling program consisted of a 95°C denaturation for 10 min, followed by 40 cycles of 95°C for 15 s and 60°C for 60 s. All samples were run in duplicate, using an automated pipetting system (epMotion M5073, Eppendorf, Hamburg, Germany), and the mean C_q_ values for each trial were calculated. Reactions with template free control were included for each set of primers on each plate.

### Data analysis

Quantification of the target mRNA was normalised using reference mRNA. *TBP*, *B2M*, and *18S rRNA* were the three most stably expressed reference genes, with similar amplification efficiencies to our target gene, out of the six reference genes assayed (Tables 5 and 6). ΔC_q_ was calculated as the difference between target and the geometric mean of our three reference genes. ΔΔC_q_ was obtained by normalising the ΔC_q_ values of the treatments to the ΔC_q_ values of the pre-treatment control.

### Statistical analysis

Kruskal-Wallis Tests were used to compare the RNA yield from different sample handling procedures and extraction methods, as well as the RNA concentration from different storage methods, using IBM SPSS Statistics V24 (IBM Corporation, Somers, NY, USA). Pearson’s correlation coefficient (r) was used to assess the relationship between RNA input and average relative amount of cDNA content measured in Quant-iT^™^ OliGreen assay using GraphPad Prism 7 (GraphPad Software, Inc., La Jolla, CA, USA). RefFinder was utilised for the statistical analysis of reference genes. A paired student’s t-test was used to compare the difference in C_q_ values, and the analyses were performed on the 2^−ΔCq^ data using Excel.

## Supporting information

S1 Table Individual data for RNA concentration and quality in Experiment 1. (PDF)

S2 Table Individual data for RNA concentration and quality in Experiment 2. (PDF)

S3 Table Individual data for RNA concentration and quality in Experiment 3. (PDF)

S4 Table Raw data for *Cyclophilin* and *PGC-1α* primer amplification efficiency test. (PDF)

S5 Table Raw data for primer efficiency test in Experiment 4. (PDF)

S6 Table Raw C_q_ value of six commonly-used reference genes in Experiment 4. (PDF)

S7 Table Raw data for determination of cDNA amount in Experiment 5. (PDF)

S8 Table Raw C_q_ value for *PGC-1α* mRNA and cDNA content in Experiment 6. (PDF)

S9 Table Raw data for correlation analysis between relative gene expression and *A*_260_/*A*_230_ ratio. (XLSX)

S1 Fig Correlation analysis between relative expression of *ACTB*, *TBP*, *Cyclophilin*, *GAPDH*, *B2M* and *18S rRNA* and *A*_260_/*A*_230_ (n = 20). ΔC_q_ is calculated as the difference of the C_q_ value between each target gene and geometric mean of other five reference genes. Individual data from each sample is presented. There was no significant correlation between expression of references genes and the *A*_260_/*A*_230_ ratio (0.4 > r > -0.4, P> 0.08). This indicates that RNA samples with a low *A*_260_/*A*_230_ ratio performed similarly in the qPCR reaction as samples with a higher ratio.
(TIF)
