# Supplementary material for: An overview of technical considerations when using quantitative real-time PCR analysis of gene expression in human exercise research
Source: PLoS One. 2018 May 10;13(5):e0196438. doi: 10.1371/journal.pone.0196438 (PMC5944930; doi:10.1371/journal.pone.0196438)
Supplement: S1 Table — (PDF) [file pone.0196438.s001.pdf]

S1 Table:
Individual data for RNA concentration and quality in Experiment 1

| Sample | RNA concentration (ng/µl) | RNA yield (ng RNA per mg muscle) | RQI | RQI classification | $A_{260}/A_{280}$ | $A_{260}/A_{230}$ |
|---|---|---|---|---|---|---|
| Good Practice 1 | 197.50 | 318.5 | 8.00 | Pass | 1.85 | 0.58 |
| Good Practice 2 | 280.50 | 480.9 | 8.10 | Pass | 1.80 | 1.22 |
| Good Practice 3 | 340.00 | 505.0 | 8.60 | Pass | 1.84 | 1.09 |
| Good Practice 4 | 278.50 | 397.9 | 9.40 | Pass | 1.80 | 1.20 |
| Thaw 1 | 185.00 | 430.2 | 7.80 | Pass | 1.68 | 1.52 |
| Thaw 2 | 215.50 | 659.7 | 7.80 | Pass | 1.74 | 0.33 |
| Thaw 3 | 268.50 | 676.9 | 8.50 | Pass | 1.79 | 1.00 |
| Thaw 4 | 119.00 | 324.5 | 8.40 | Pass | 1.56 | 1.29 |
| Freee thaw 1 | 225.50 | 697.4 | 8.20 | Pass | 1.75 | 0.96 |
| Freee thaw 2 | 166.50 | 675.0 | 8.20 | Pass | 1.69 | 0.38 |
| Freee thaw 3 | 259.50 | 949.4 | 9.00 | Pass | 1.77 | 0.80 |
| Freee thaw 4 | 246.50 | 859.9 | 8.90 | Pass | 1.84 | 1.34 |
