# Supplementary material for: An overview of technical considerations when using quantitative real-time PCR analysis of gene expression in human exercise research
Source: PLoS One. 2018 May 10;13(5):e0196438. doi: 10.1371/journal.pone.0196438 (PMC5944930; doi:10.1371/journal.pone.0196438)
Supplement: S2 Table — (PDF) [file pone.0196438.s002.pdf]

S2 Table:
Individual data for RNA concentration and quality in Experiment 2

Sample	RNA concentration (ng/µl)	RNA yield (ng RNA per mg muscle)	RQI	RQI classification	A_{260}/A_{280}	A_{260}/A_{230}
Trizol 1	308.00	616.0	9.30	Pass	1.68	0.51
Trizol 2	392.00	683.7	8.00	Pass	1.77	0.37
Trizol 3	542.00	1084.0	9.10	Pass	1.80	0.27
Trizol 4	318.50	682.5	8.80	Pass	1.70	0.52
Kit + 2-propanol 1	197.50	318.5	8.00	Pass	1.85	0.58
Kit + 2-propanol 2	280.50	480.9	8.10	Pass	1.80	1.22
Kit + 2-propanol 3	340.00	505.0	8.60	Pass	1.84	1.09
Kit + 2-propanol 4	278.50	397.9	9.40	Pass	1.80	1.20
Kit + Ethanol 1	30.50	59.0	4.20	Not Passed	1.13	0.60
Kit + Ethanol 2	40.50	122.7	8.90	Pass	1.07	0.29
Kit + Ethanol 3	22.00	71.0	9.20	Pass	0.75	0.06
Kit + Ethanol 4	24.00	98.6	8.40	Pass	0.77	0.34
