# Supplementary material for: An overview of technical considerations when using quantitative real-time PCR analysis of gene expression in human exercise research
Source: PLoS One. 2018 May 10;13(5):e0196438. doi: 10.1371/journal.pone.0196438 (PMC5944930; doi:10.1371/journal.pone.0196438)
Supplement: S3 Table — (PDF) [file pone.0196438.s003.pdf]

S3 Table 3:

Individual data for RNA concentration and quality in Experiment 3

Sample	RNA concentration (ng/µl)	RQI	RQI classification	A_{260}/A_{280}	A_{260}/A_{230}
Intact RNA 1	197.5	8.00	Pass	1.85	0.58
Intact RNA 2	280.5	8.10	Pass	1.80	1.22
Intact RNA 3	340.0	8.60	Pass	1.84	1.09
Intact RNA 4	278.5	9.40	Pass	1.80	1.20
Degraded RNA 1	236.5	7.90	Pass	2.18	0.59
Degraded RNA 2	330.0	8.40	Pass	1.97	1.15
Degraded RNA 3	369.0	8.30	Pass	1.90	1.01
Degraded RNA 4	311.0	8.80	Pass	1.87	1.11
RNase treated 1	96.0	N/A	Not Passed	1.86	0.33
RNase treated 2	128.5	N/A	Not Passed	1.86	1.56
