# Supplementary material for: An overview of technical considerations when using quantitative real-time PCR analysis of gene expression in human exercise research
Source: PLoS One. 2018 May 10;13(5):e0196438. doi: 10.1371/journal.pone.0196438 (PMC5944930; doi:10.1371/journal.pone.0196438)
Supplement: S4 Table — (PDF) [file pone.0196438.s004.pdf]

S4 Table:

Raw data for Cyclophilin and PGC-1α primer amplification efficiency test

	C_q Value					Slope	Effciency	Effciency %	R^2
cDNA copy	1	0.1	0.01	0.001	0.0001				
Log (cDNA copy)	0	-1	-2	-3	-4				
Cyclophilin	21.70	24.77	28.35	32.22	34.54	-3.31	2.00	100.44	0.99
PGC-1 α	20.46	23.37	27.28	30.72	32.90	-3.22	2.04	104.37	0.99
Δ C_q	1.24	1.40	1.07	1.50	1.64	-0.09			
