# Supplementary material for: An overview of technical considerations when using quantitative real-time PCR analysis of gene expression in human exercise research
Source: PLoS One. 2018 May 10;13(5):e0196438. doi: 10.1371/journal.pone.0196438 (PMC5944930; doi:10.1371/journal.pone.0196438)
Supplement: S5 Table — (PDF) [file pone.0196438.s005.pdf]

S5 Table:
Raw data for primer efficiency test in Experiment 4

	Cq					Slope	Effciency	Effciency %	R^2
cDNA copy	1	0.1	0.01	0.001	0.0001				
Log (cDNA copy)	0	-1	-2	-3	-4				
GAPDH	18.37	20.52	24.28	27.27	30.92	-3.19	2.06	106.01	0.99
B2M	19.03	21.58	25.30	28.81	32.25	-3.37	1.98	98.15	1.00
Cyclophilin	21.70	24.77	28.35	32.22	34.54	-3.31	2.00	100.44	0.99
18s rRNA	9.15	11.94	15.60	19.02	22.33	-3.34	1.99	99.12	1.00
PGC-1α	20.46	23.37	27.28	30.72	32.90	-3.22	2.04	104.37	0.99

	Cq					Slope	Effciency	Effciency %	R^2
cDNA copy	1	0.5	0.25	0.125	0.0625				
Log (cDNA copy)	0	-0.301	-0.6021	-0.9031	-1.2041				
ACTB	27.30	28.30	29.41	30.53	30.96	-3.17	2.07	106.71	0.98
TBP	27.34	28.44	29.69	30.70	31.25	-3.34	1.99	99.09	0.98
