# Supplementary material for: An overview of technical considerations when using quantitative real-time PCR analysis of gene expression in human exercise research
Source: PLoS One. 2018 May 10;13(5):e0196438. doi: 10.1371/journal.pone.0196438 (PMC5944930; doi:10.1371/journal.pone.0196438)
Supplement: S6 Table — (PDF) [file pone.0196438.s006.pdf]

S6 Table:

Raw C_q value of six commonly-used reference genes in Experiment 4

TBP	Timepoints (Hours)		
Participant	Baseline	0	3
1	28.55	26.40	25.53
2	25.11	25.93	25.84
3	25.21	25.14	25.56
4	25.90	26.94	26.35
5	26.58	26.87	25.84
6	25.76	25.56	26.27
7	27.88	26.80	28.15
8	26.84	26.67	26.00
9	26.08	26.64	26.24

ACTB	Timepoints (Hours)		
Participants	Baseline	0	3
1	26.09	23.95	22.37
2	22.85	23.01	21.77
3	22.74	21.43	22.13
4	23.02	23.41	23.98
5	24.19	24.39	23.38
6	23.24	22.82	23.10
7	24.95	23.74	23.61
8	23.86	22.80	23.07
9	24.44	23.75	23.70

**B2M**	Timepoints (Hours)		
Participant	Baseline	0	3
1	24.50	22.23	21.40
2	21.29	20.83	20.52
3	20.98	20.35	20.58
4	21.37	21.09	21.63
5	21.09	21.43	21.14
6	21.54	21.08	20.70
7	22.55	21.78	22.08
8	21.40	20.73	20.65
9	21.39	21.47	21.12

Cyclophilin	Timepoints (Hours)		
Participants	Baseline	0	3
1	30.58	28.36	28.22
2	26.05	27.19	26.37
3	27.29	26.18	26.42
4	26.42	28.48	28.32
5	27.76	27.87	28.09
6	26.41	26.71	27.22
7	28.87	29.26	29.65
8	27.50	28.11	27.84
9	27.92	28.25	28.56

GAPDH	Timepoints (Hours)		
Participant	Baseline	0	3
1	23.29	21.45	19.91
2	19.28	18.97	20.47
3	18.91	19.18	19.08
4	20.33	20.88	21.04
5	21.63	21.82	20.49
6	20.33	20.83	21.45
7	22.34	21.07	22.70
8	20.78	20.67	20.09
9	20.41	19.83	21.33

18S	Timepoints (Hours)		
Participants	Baseline	0	3
1	15.70	13.20	12.28
2	11.80	11.34	12.30
3	11.08	11.91	11.00
4	12.24	12.57	13.27
5	12.56	13.01	11.83
6	11.76	11.98	11.89
7	13.52	12.58	13.53
8	12.21	11.71	11.77
9	12.63	10.89	12.37
