# Supplementary material for: An overview of technical considerations when using quantitative real-time PCR analysis of gene expression in human exercise research
Source: PLoS One. 2018 May 10;13(5):e0196438. doi: 10.1371/journal.pone.0196438 (PMC5944930; doi:10.1371/journal.pone.0196438)
Supplement: S7 Table — (PDF) [file pone.0196438.s007.pdf]

S7 Table:
Raw data for determination of cDNA amount in Experiment 5

RNA input (µg)	cDNA content (A.U.)	Mean	SD
1	2.97	2.90	0.10
1	2.90		
1	2.77		
1	2.97		
0.5	2.01	1.98	0.08
0.5	2.07		
0.5	1.96		
0.5	1.89		
0.25	1.57	1.44	0.10
0.25	1.38		
0.25	1.35		
0.25	1.46		
0	1.06	1.01	0.03
0	0.99		
0	1.01		
0	0.98		
1 (-RT)	2.64	2.55	0.14
1 (-RT)	2.71		
1 (-RT)	2.42		
1 (-RT)	2.45		
