# Supplementary material for: An overview of technical considerations when using quantitative real-time PCR analysis of gene expression in human exercise research
Source: PLoS One. 2018 May 10;13(5):e0196438. doi: 10.1371/journal.pone.0196438 (PMC5944930; doi:10.1371/journal.pone.0196438)
Supplement: S8 Table — (PDF) [file pone.0196438.s008.pdf]

S8 Table:

Raw C_q value for *PGC-1α* mRNA and cDNA content in Experiment 6

C_q value for *PGC-1α*

PGC-1 α	Timepoints (Hours)		
Participants	Baseline	0	3
1	24.41	22.54	20.08
2	21.46	21.58	20.02
3	22.13	21.92	19.04
4	22.44	22.96	21.05
5	23.12	23.41	20.95
6	21.68	21.85	21.92
7	24.37	23.83	22.19
8	23.35	24.15	21.33
9	22.06	23.65	20.86

cDNA content measured by OliGreen dye

cDNA content	Timepoints (Hours)		
Participants	Baseline	0	3
1	0.27	1.27	2.42
2	3.39	3.17	3.73
3	3.63	3.46	3.86
4	2.41	2.05	2.61
5	2.71	2.90	2.80
6	2.90	2.86	2.35
7	1.48	2.43	2.40
8	1.75	2.17	2.46
9	3.30	3.55	3.67
