# Supplementary figures and images for: An overview of technical considerations when using quantitative real-time PCR analysis of gene expression in human exercise research
Source: PLoS One. 2018 May 10;13(5):e0196438. doi: 10.1371/journal.pone.0196438 (PMC5944930; doi:10.1371/journal.pone.0196438)

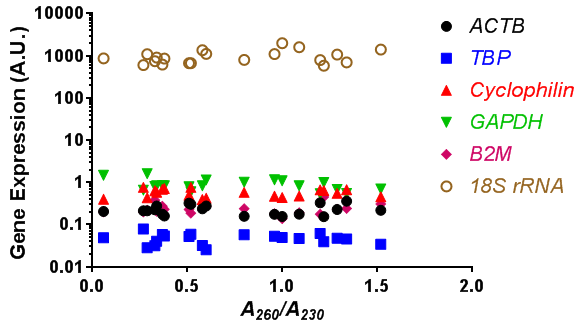

Supplement: S1 Fig — ΔCq is calculated as the difference of the Cq value between each target gene and geometric mean of other five reference genes. Individual data from each sample is presented. There was no significant correlation between expression of references genes and the A260/A230 ratio (0.4 > r > -0.4, P> 0.08). This indicates that RNA samples with a low A260/A230 ratio performed similarly in the qPCR reaction as samples with a higher ratio. (TIF) [file pone.0196438.s010.tif]
